# Modelling of Hardness and Electrical Conductivity of Cu-4Ti (wt.%) Alloy and Estimation of Aging Parameters Using Metaheuristic Algorithms

**DOI:** 10.3390/ma18102366

**Published:** 2025-05-19

**Authors:** Jarosław Konieczny, Krzysztof Labisz, Satılmış Ürgün, Halil Yiğit, Sinan Fidan, Mustafa Özgür Bora, Şaban Hakan Atapek, Janusz Ćwiek

**Affiliations:** 1 Department of Railway Transport, Faculty of Transport and Aviation Engineering, Silesian University of Technology, Krsińskiego 8 Street, 40-019 Katowice, Poland; jaroslaw.konieczny@polsl.pl (J.K.); janusz.cwiek@polsl.pl (J.Ć.); 2 Faculty of Aviation and Astronautics, Aviation Electrical Electronics, Kocaeli University, İzmit 41001, Kocaeli, Türkiye; urgun@kocaeli.edu.tr; 3 Department of Information Systems Engineering, Kocaeli University, İzmit 41001, Kocaeli, Türkiye; halilyigit@kocaeli.edu.tr; 4 Faculty of Aviation and Astronautics, Airframe and Powerplant Maintenance, Kocaeli University, İzmit 41001, Kocaeli, Türkiye; sfidan@kocaeli.edu.tr (S.F.); ozgur.bora@kocaeli.edu.tr (M.Ö.B.); 5 Laboratory of High Temperature Materials, Faculty of Engineering, Department of Metallurgical and Materials Engineering, Kocaeli University, İzmit 41001, Kocaeli, Türkiye; hatapek@kocaeli.edu.tr

**Keywords:** Cu-4Ti alloy, precipitation hardening, cold deformation, electrical conductivity, hardness, metaheuristic optimization, microstructural evolution, alloy design

## Abstract

This study focuses on cold deformation and age effects on the microhardness and electric conductivity of the Cu-4Ti (wt.%) alloys. The samples were solution treated at 900 °C, quenched in water, and aged at 450–600 °C for 1–120 min. Fifty percent cold rolling was performed before aging to analyze the impact on their microstructure and properties. Hardness and electric conductivity were examined by the Vickers microhardness and Förster testing. Hardness increased significantly while electric conductivity was maintained. The optimal hardness of 298 HV appeared following 50% cold rolling and aging for 120 min at 450 °C, and an electric conductivity of 9.4 MS/m was achieved after 120 min at 600 °C in cold-rolled materials. The deformed and solution-treated materials reached 244 HV after 120 min at 500 °C, and electric conductivity reached 7.7 MS/m. Polynomial models of regression were used to analyze the impact of aging parameters on properties. Process parameters were properly optimized by applying metaheuristic algorithms. These contributions ensure a better understanding of the relationship between the microstructure and properties in Cu-Ti alloys, as well as their application in aircraft and electronics.

## 1. Introduction

Even after strengthening by way of alloying or heat treatment, copper alloys maintain good electrical conductivity, and this makes copper alloys perfect for electrical applications, including cables and connectors. Although strengthening by alloying, precipitation, and grain refinement is normally practiced, in most treatments, electrical conductivity is typically compromised. Among these treatment processes, precipitation hardening is most effective for maintaining an optimal tradeoff between electrical performance, strength, and cost, while being required for industrial application. When efficiently applied, this technique guarantees reliable and cost-effective materials with good conductivity [1]. High-performance copper alloys must maintain good electrical and thermal conductivity in addition to better mechanical properties. During aging treatment, precipitation hardening successfully enhances these qualities. By reducing conduction electron scattering, the solute content of alloying elements is essential for preserving good electrical conductivity. As a result, strategic application of precipitation hardening is a crucial strengthening mechanism, ensuring that copper alloys satisfy the exact specifications of present and future automotive and electronic industry applications [2]. Because of their stable electrical characteristics, copper alloys, such as manganin (13.5% Cu–Mn–4% Ni), are frequently used as precision resistors for low-current wires at low temperatures. After 60% cold deformation of as-cast samples and 30 min of annealing at 400 °C, a hardness of 200 HV and 30% IACS conductivity may be achieved. But there are several applications where such hardness is inadequate. Titanium (Ti) is age-hardenable, and copper dissolves up to 9 wt.%. Ti can be added to the alloy to enhance its hardness while maintaining electrical conductivity [3]. Cu-Ti alloys have attracted considerable interest on account of their good age-strengthening behavior. For improved quality of their alloys, attempts have been made to optimize their parameters and composition through studies. The residence of Ti solutes in the solid solution matrices of Cu-Ti alloys creates deformation in their lattices, arresting the migration of dislocation. Precipitation of a metastable β′ phase, which is formed over the course of aging and greatly improves mechanical properties while preserving the desired performance characteristics, is the main strengthening process [4]. Ti makes Cu-Ti alloys harder but lowers their electrical conductivity. Contemporary Cu-Ti alloys have achieved electric conductivity in excess of 60% IACS and hardness exceeding 400 HV. However, it is challenging to excel in both electrical conductivity and hardness. For example, electrical conductivity is lowered to approx. 10% IACS if the hardness of Cu-Ti alloys exceeds 300 HV, indicating a compromise between such behavior and the optimal design of the alloys [5].

A popular method for improving the mechanical characteristics and microstructure of metallic materials is work hardening. By producing sub-micron or nanocrystalline grain patterns using severe plastic deformation (SPD), one can greatly increase the room-temperature strength and high-temperature ductility of copper alloys. Numerous SPD routes have evolved, including accumulative roll-bonding, equal-channel angular pressing, groove rolling, high-pressure torsion, and slope extrusion [6,7,8,9,10]. Nonetheless, they have persistent shortcomings, including an inability to obtain reproducible microstructures in large product volumes, poor edge quality, and low bonding strength between interfaces following accumulative roll-bonding [11]. Cold working lowers malleability and deformability while raising tensile strength, hardness, surface quality, dimensional accuracy, and workability. The work hardening method which has been applied in practice most extensively, namely cold rolling, strengthens metals by minimizing their grain sizes up to a limit. Minimizing the crystal structure’s dislocation using a heat treatment technique, which is termed as annealing, restores its ductility and lowers its hardness. Annealing, typically applied after cold working, improves formability and machinability, reduces residual stress, and prevents brittleness failure, which leads to better handling and durability of the material [12]. Cu-Ti alloys strengthen by means of Cu_4_Ti precipitates of an intermetallic compound in the Cu matrix upon aging. Cu-3.5 wt% Ti (as well as Cu-4.6 wt% Ti) alloys, subject to homogeneous and inhomogeneous deformation, were aged at 450 °C following solution treatment at 885 °C. Inhomogeneously deforming alloys formed shear bands, hence their increased hardness and conductivity compared to undeforming and homogeneously deforming alloys featuring slip bands. Peak hardness developed earlier in the shear-banded (30 min) specimens compared to the slip-banded (720 min) and undeforming (1440 min) ones. The optimal 16.8% IACS and 302 HV combination developed after 360 min of aging [13]. High-strength Cu–Ti–Fe alloys were synthesized, and their microstructural development and property improvement were examined. Sixty percent cold deformation and subsequent 450 °C/240 min aging increased the microhardness and conductivity of Cu-1.5Ti-0.3Fe to 269.3 HV and 19.8% IACS, compared to 196.8 HV and 7.8%, respectively. The principal precipitates, Fe_2_Ti and Cu_4_Ti, retard dislocation movement and recrystallization, thus contributing to the strengthening mechanisms. The grain boundary solution and deformation strengthening improve the alloy’s electrical conductivity by increasing the content of solute atoms [14]. The structure and properties of the solution as well as of the cold-worked and aged Cu-4.5% Ti alloy were studied by microscopy, microhardness, tensile, and electrical properties testing. Prior cold working increased peak microhardness to 425 VHN, tensile strength to 1380 MPa, and electrical conductivity to 25% IACS, compared to 340 VHN, 890 MPa, and 10%, respectively, after aging at 400–450 °C. The strengthening effect is attributed to the metastable, coherent precipitates of the β′ phase (Cu4Ti). Abundant profuse twinning facilitates deformation, and prior cold working does not influence the fracture mode of the alloy [15]. Using hardness, tensile, and microscopy tests, the effects of cold rolling to 50%, 75%, and 90% reductions on the age-hardening behavior of the Cu–4Ti–1Cr alloy were examined. Hardness increased to 416 HV (90% cold work with peak aging) from 222 HV (solution-treated). The peak aging temperature decreased from 450 °C (undeformed) to 400 °C due to cold deformation. The alloy demonstrated a yield strength of 1165 MPa and an ultimate tensile strength of 1248 MPa after 90% cold work and peak aging at 400 °C. Due to the formation of the incoherent β′ phase (Cu_3_Ti), over-aging resulted in lower hardness and strength [16]. Wang et al. [17] investigated the evolution of the microstructure and properties of the Cu-2.7Ti-0.2Fe alloy during cold drawing and subsequent aging. Grain refinement mechanisms were analyzed through electron back scattered diffraction (EBSD), revealing dislocation evolution and twin–twin/dislocation intersections as key contributors. Cold drawing induced strong <100> and <111> fiber textures, with volume fractions of 24.9% and 55.5%, respectively, at a true strain of 8.4. The <100> fiber texture was linked to twinning and dislocation slipping in high Schmid factor regions. After peak aging at 380 °C for 2 h, the alloy exhibited excellent properties: a yield strength of 1596 MPa, a tensile strength of 1682 MPa, and an electrical conductivity of 15.5% IACS. The Cu–Ti alloys were synthesized using the accumulative roll bonding–deformation diffusion (ARB-DD) process to study their deformation-aging behavior, microstructural evolution, and properties. The process led to the formation of multiscale substructures like Taylor lattices, which facilitated the homogeneous nucleation of the coherent Cu_4_Ti particles during aging. These particles interacted with dislocations, significantly enhancing their strength. The ultimate tensile strength (UTS) reached 1040 MPa, representing a 160% improvement, while electrical conductivity increased by 90%, reaching 13% IACS. The Cu_4_Ti particles and reduced low-angle grain boundaries (LAGBs) contributed to these enhancements by reducing electron scattering [18]. The transformation kinetics and phase equilibrium of both metastable and stable precipitates in the Cu-4 at. pct Ti alloy were studied during isothermal aging at 693–973 K (420–700 °C). The microstructure evolved over four stages: spinodal decomposition, continuous precipitation of tetragonal β′-Cu_4_Ti needles, discontinuous precipitation of orthorhombic β-Cu_4_Ti lamellae, and equilibrium saturation. Below 923 K, β-Cu_4_Ti forms via a cellular reaction, while at 973 K, it also derives from β′-Cu_4_Ti. A TTT diagram and a Cu–Ti phase diagram elucidated the correlations between the microstructure, strength, and conductivity [19]. The effect of aging on the microstructure and properties of the cold-rolled Cu–3Ti–2Mg alloy was investigated. The alloy comprises β′-Cu_4_Ti, Cu_2_Mg, and a Cu matrix. The cold rolling elongated the grains and homogenized the microstructure, promoting the β′-Cu_4_Ti precipitation during aging. Prolonged aging transformed β′-Cu_4_Ti into β′-Cu_3_Ti. Optimal properties were achieved after 70% deformation and aging at 450 °C for 3 h, yielding a hardness of 357.6 HV, a conductivity of 18.5% IACS, and a tensile strength of 401 MPa [20]. Eze et al. [21] examined the electrical conductivity and mechanical properties of pure Cu and Cu-Ti alloys (CuTi0.014 and CuTi0.035 with 1 and 2.6 mass% Ti) within the Cu–solid solution region. Samples were prepared by mixing with alumina balls and sintered at 650 °C under a 50 MPa load. CuTi0.035 exhibited the highest hardness (749 MPa), yield strength (1604 MPa), and ultimate tensile strength (1318 MPa), while CuTi0.014 achieved superior electrical conductivity (5.0 S/m at 550 °C) and balanced properties. Titanium additions enhanced corrosion resistance and friction coefficients under dry sliding. CuTi0.014 is recommended for high-temperature applications requiring excellent mechanical and electrical performance. Through microalloying, deformation, and aging, a Cu-3.3 wt.% Ti alloy was able to obtain high strength (1118 MPa) and electrical conductivity (21% IACS). By adding 0.1 wt.% Mg and using the preaging + cold rolling + aging method, the dislocation distribution uniformity was improved and the precipitate’s size decreased, while multiscale precipitate formation with a higher volume fraction was encouraged in comparison to Mg-free alloys. Strength and conductivity were enhanced as the dislocations and precipitates were working in concert. These results shed light on the mechanisms behind Cu-Ti alloys and imply recommendations for the industrial manufacturing of high-performance Cu-Ti alloys [22].

The alloy’s microstructure and processing conditions, such as solution treatment, aging, and cold working, affect the CuTi alloy’s behavior. Since composition and processing conditions interact in such a multifaceted way, designing alloys to satisfy specified criteria is still challenging. Computation tools such as machine learning, thermodynamic and phase-field simulations, and ab initio computations provide alternative strategies for designing rapidly. The design of Cu alloys has been supported by machine learning in general. However, its applicability to Cu-Ti alloys is still unknown, yet it provides an avenue for future studies in this context [4]. Innovation is restricted by the time-consuming and costly nature of traditional trial-and-error alloy design techniques. A revolutionary method for deciphering intricate correlations in experimental datasets and accelerating alloy development is machine learning (ML). An ML-driven approach to identify high-performance Cu alloys from rejected experimental data with less-than-ideal electrical conductivity or hardness has been presented. Regression models for the Gaussian process, improved by further characteristics, predicted alloys with better qualities, which were then confirmed experimentally. Second-phase Co-Ti and Fe-Ti precipitates produced improved hardness, and matrix purification produced improved conductivity. The potential of machine learning in alloy design was highlighted by the additional evaluation of feature relevance through genetic algorithms [23]. By reducing trial-and-error experimentation and speeding up the discovery of new materials, ML transformed materials research altogether. To design a composition–process–property strategy, Guo et al. [24] applied ML to screen for improved property alloys based on principal component alloying element descriptors and conventional ML techniques. As anticipated, the XGBoost model selected the Cu-3Ti-0.3Cr-0.15Mg alloy, its ultimate tensile strength and IACS conductivity being 1018 MPa and 20.1%, respectively. The formation of nanoscale β′-Cu_4_Ti and Cr precipitates during heat treatment was held responsible for its improved strength. The potential of ML in the design of Cu-Ti alloys and other material systems has been demonstrated. Li et al. [25] developed an XGBoost-based ML model to predict Cu alloy characteristics after testing regression techniques on a few datasets. Process parameters were incorporated to create a composition–process–property relationship. Alloy systems characterized by enhanced strength, conductivity, and ductility can be effectively discovered using a three-step methodology for designing alloys, including property extraction, error screening, and expected improvement (EI) calculation. The Cu-Ni-Co-Si-Mg and Cu-Ni-Co-Si-Zn alloys display good elongation, electrical conductivity, and ultimate strength in tension. Cu-Ni-Si alloys and other materials with conflicting characteristics and wide design spaces can be efficiently optimized using this technology. Simulations based on density functional theory (DFT) and molecular dynamics (MD) with modified embedded atom method (MEAM) potentials were used to examine the mechanical characteristics of Cu-Ti alloys. By raising the local charge density, Ti increases the tensile stress in bicrystalline Cu, as shown by DFT calculations. MEAM simulations revealed that the addition of Ti decreased the density of stacking faults and prevented the formation of partial Shockley dislocations at Cu grain boundaries. Polycrystalline Cu showed enhanced yield strength and elastic modulus even at 1.5 wt.% Ti. These results clarify the function of Ti in fortifying Cu and directing grain boundary engineering for improved performance [26]. Babaheydari et al. [27] developed a feed-forward backpropagation neural network (FFBPNN) to predict the hardness of copper-based nanocomposites. Nanocomposites, including one, three, and six weight percentages of Cu-Al, Cu-Al_2_O_3_, Cu-Cr, and Cu-Ti, were synthesized through mechanical alloying, compacted under 12 tons, and 650 °C heat treatment. The reinforcement property, vial speed, and weight ratio of ball/powder and milling time were parameters for the neural network, while hardness was taken as an output. The optimized model, featuring two hidden layers (12 and 8 neurons), achieved a regression of 0.9914 and an RMSE of 3.7%, accurately predicting micro-hardness. The effect of Ti additions on the microstructure and properties of Cu-Cr alloys was studied, establishing a processing–microstructure–property relationship using the CALPHAD (calculation of phase diagram)-based integrated computational materials engineering (ICME) approach, which was validated experimentally. The peak-aged 0.5Cu-0.2Ti-Cr alloy achieved excellent values: tensile strength of 629.3 MPa, yield strength of 603.0 MPa, 192.6 HV, electrical conductivity of 51.3% IACS, and 10.9% elongation. The addition of Ti favored Cr precipitate nucleation and inhibited Cr precipitate growth, giving rise to nano-sized Cr precipitates upon aging. The microstructural analysis confirmed the foregoing, providing evidence for the capability of the ICME flowchart in designing compositions and optimizing processes for engineering applications [28].

Metaheuristic algorithms have become an important research topic in the fields of engineering, optimization, and artificial intelligence in recent years. Inspired by nature, physics, biology, and social systems, these algorithms provide high efficiency and flexibility in solving complex problems. Especially in engineering applications, where many parameters need to be optimized, they can provide better solutions by overcoming the limitations of traditional methods. An Artificial Hummingbird Algorithm (AHA) inspired by the flight abilities and feeding strategies of hummingbirds is proposed, and its performance is investigated with regard to optimization problems. Evaluations on different test functions and engineering problems have shown that the AHA offers a low computational load and high solution accuracy. Furthermore, its implementation in real-life contexts, such as hydraulic power operation design, validated the viability of using the algorithm in real-life applications [29]. The Ant Lion Optimizer (ALO) is a nature-inspired optimization algorithm that mimics the hunting mechanism of ant lions to solve complex optimization problems efficiently. The research on the application of ALO, including this study, clearly demonstrates its capability to optimize prediction modeling hyperparameters. Evidence shows how enhanced prediction of the punching shear strength of FRP-RC beams can be performed using the ALO-RF model compared to other models applied in testing and training phases [30]. Atom Search Optimization (ASO) is a metaheuristic optimization algorithm inspired by physical molecular dynamics; it seeks optimal solutions in the search space by representing the Lennard–Jones potential and the effect of the bond length of atoms on the mathematical model. In this study, a variant of ASO integrated with chaotic maps (CASOs) exhibited superior performance in ARX-based model identification of electro-hydraulic actuator systems (EHASs) with low mean square error (MSE) compared to conventional ASO and other metaheuristics. In particular, CASO5, developed with a logistic chaotic map, proved the effectiveness of ASO-based approaches against industrial control problems by providing consistency and reliability in modeling the nonlinear dynamics of the system [31]. In this study, Atom Search Optimization (ASO), a physics-based optimization method, is enhanced with a proposed hybrid approach (HASO) for the repositioning of cross-boundary atoms and effectively applied to the design of a nonlinear fractional-ordered PID (NL-FOPID) controller, providing significant improvements in convergence speed and optimization performance compared to conventional methods such as GA, SA, and PSO. Designed with HASO, the controller produced results that outperformed conventional methods in a highly nonlinear continuous stirred tank reactor (CSTR) system, and it was emphasized that ASO-based hybrid algorithms have a wide application potential in engineering problems [32]. The improved flow direction approach (LSRFDA) using the combination of a Lévy flight and the self-renewable approach is superior in search efficiency and local minimum avoidance than basic FDA. Studies exhibit enhanced performance of LSRFDA against benchmark problems and engineering optimization, and thus in future industrial practices [33]. Nineteen metaheuristic strategies for selective harmonic elimination in multilevel inverters have been subject to a comparative analysis, and FDA has been found among the strategies capable of providing optimal THD minimization according to the standard of IEEE 519. Studies have identified FDA’s capability to accurately calculate switching angles and thus to provide enhanced voltage quality and convergence performance [34]. Moth-Flame Optimization (MFO) is a metaheuristic approach developed based on moths’ navigation behavior. Its power lies in robustness and simplicity in exploration, however, it is prone to premature convergence. In their study, Yu et al. have presented EQDXMFO, an upgraded version of MFO, by including a quality enhancement approach and a directional crossover operation. EQDXMFO demonstrates superior capability in tackling engineering constraints while analyzing engineering problems [35]. Recent comparative studies on selective harmonic elimination using metaheuristic algorithms proved the Moth-Flame Optimization (MFO) approach to be efficient, showing good convergence and efficient elimination of harmonics while maintaining total harmonic distortion (THD) to a minimum under strict industry constraints.

The power of MFO lies in good trade-offs between exploration and exploitation; hence, it avoids local optima and produces good quality voltage waveforms, and is therefore found to be a general and robust solution for solving complex harmonic elimination problems in power electronic systems [36]. The Marine Predator Algorithm (MPA) is inspired by optimal foraging behavior of marine predators and applies Lévy and Brownian movements for efficient search. An advanced version of MPA known as DOTMPA has been proposed in the paper, combining dynamic opposition-based learning and a Taylor optimal neighborhood approach to ensure better global convergence and search capability [37]. The comprehensive opposition multi-verse optimizer (COMVO) is an advanced version of the basic Multi-Verse Optimizer (MVO), featuring better exploration mechanisms and applying a specific technique to constraints, which has been found to provide better solutions to difficult scheduling problems [38]. A metaheuristic-based process is proposed to optimize the notch width and depth in terms of laser power, cutting speed, and nozzle distance parameters during carbon dioxide laser cutting of polymethylmethacrylate sheets. Four different metaheuristic methods, such as the genetic algorithm, particle swarm optimization, the whale optimization algorithm, and ant lion optimization, are applied to the problem of generating the optimum notch geometry. While all methods are efficient and accurate enough, the PSO method is arguably the most cost-effective one [39]. SSO (Salp Swarm Optimization) also stands out as an effective metaheuristic algorithm that balances exploration and exploitation with a single parameter, and which has been successfully applied in various fields, such as machine learning and engineering design; it offers an attractive option for researchers, especially with advantages such as hybridisability and low computational complexity [40]. Experimental methods and metaheuristic algorithms were used to optimize the CuNi2Si1 alloy’s mechanical and electrical behavior. Hardness and electrical conductivity were explored by applying aging temperature (450–600 °C) and time (1–420 min) variations. Cold rolling under 50% strain following solution annealing purified the microstructure and promoted Ni2Si precipitation. 266 HV and 13 MS/m were the values obtained in undeformed samples under the best conditions of 450 °C and 30 min. SPBO outperformed GA and PSO in terms of prediction accuracy, predicting 450 °C for 61.75 min, while experimental confirmation demonstrated 267 HV and 14 MS/m [41]. High-entropy alloys (HEAs) occupy an enormous compositional space and make the design of high-performance materials arduous. Poonia et al. [42] suggested an algorithmic platform for hardness maximization through alloy composition optimization. The phase effect on hardness prediction was explored thoroughly. A generalized framework disregarding the elements of concern was achieved by integrating HEA classification, multi-label phase prediction, and hardness optimization. The framework predicts the optimal composition by using 29 descriptors of the alloy as well as its phases, based on the processing route, and whether the alloy is an HEA, thus enhancing the designing efficiency of the material. Kolev [43] developed a model used to predict the mechanical properties of Cu-Ti alloys based on a dataset of more than 1000 data points, comprising compositional elements and processing parameters. The elements in question were Cu, Al, Ce, Cr, Fe, Mg, Ti, and Zr, in conjunction with thermo-mechanical characteristics. A random forest regressor, refined by GridSearchCV, demonstrated exceptional predictive accuracy with R^2^ values of 0.9929, 0.9851, and 0.9937 for the training, validation, and test datasets, respectively. The criterion of property importance and a correlation heatmap were used to determine the contribution of processing parameters and alloy composition on mechanical properties. The complexity of HSR, being a complex process, makes it challenging to determine the optimum process conditions due to the slab width variability, seasonal impact, and dynamic operational changes. Finishing mills in HSR represent multi-stage and multi-objective optimization processes, and as such, they require accurate control of important parameters in order to achieve the desired strip thickness. Sikdar and Mukherjee [44] presented a critical review of HSR modeling and optimization with specific reference to roll force, roll profile, and cooling systems. The literature highlights the application of artificial neural networks (ANNs) for process prediction, while genetic algorithms (GAs) are widely applied for profile optimization. Previous studies of Cu-Ti alloys have revealed that precipitation hardening and cold deformation significantly influence their mechanical and electrical properties, with a challenging trade-off between enhanced hardness and reduced electrical conductivity. While extensive research efforts have been expended in pursuit of optimum hardness–conductivity trade-offs by altering deformation and aging conditions, optimization using advanced metaheuristic algorithms has been investigated less, in comparison. This research gap is addressed by the contemporary studies on the integration of polynomial regression modeling with metaheuristic algorithms to optimize the aging parameters of Cu-4Ti alloys more precisely and to maximize hardness and electrical conductivity at the same time. Not only is the microstructure-property relationship refined in this study, but a practical and transferable tool has also been presented, which makes the paper worthy of publication in a premier journal on the field of aerospace and electronics.

This study explores the effects of cold deformation after solution treatment on the electrical conductivity and hardness of a precipitation-heat-treatable Cu-4Ti alloy in terms of multiple age conditions and duration times. Following a metaheuristic approach, the study systematically investigates how such parameters affect microstructural development and the resultant property of the alloy. The experimental confirmation and the computational simulation undertaken under this study not only clarify the connection between properties and processing parameters but also offer a systematic methodology for optimizing such parameters using metaheuristic approaches. The result illustrates that combinations of age conditions and cold deformation have effects on precipitate distribution and precipitate uniformity, as well as on electrical conductivity and hardness. Such insights offer a deeper understanding of the grain boundary engineering and precipitation mechanisms in Cu-Ti alloys. This research offers significant contributions to the materials science by filling a literature gap which calls for urgent attention: lack of a systematic methodology for optimizing microstructure–processing–property relationships in Cu-Ti alloys. The novel methodology proposed in this study represents an efficient and pragmatic approach to designing copper alloys for property improvement, enabling their expanded application in the automotive, aerospace, and electronic industries. Such a metaheuristic approach to optimization is also general in nature, and as such, it can be transferred to any system of alloys, evidencing its flexibility and capacity to advance the materials science.

## 2. Experimental Procedure

### 2.1. Material Production and Determination of Properties

The initial material was an ingot of the Cu-4Ti alloy, hot-rolled to a thickness of 3.0 mm. Its chemical composition is provided in Table 1.

Following surface cleaning (etching, rinsing, and drying), hot-rolled strips were cut into samples (3 × 25 × 30 mm in size) and designated for further testing. For each condition (solution heat treatment, cold rolling, and aging), 10 samples were produced. The samples were subjected to further processing according to the following scheme:

- Variant I: Samples were heated at 900 °C for 1 h in an electric resistance furnace chamber. Once removed from the furnace chamber, the heated samples were intensively cooled in water. The time between removing the samples from the furnace and immersion in water was up to 2 s. After cooling, some samples were designated for testing of properties following solution heat treatment, and the remaining part was aged at 450, 500, 550, and 600 °C for 1, 5, 15, 30, 60, 120, and 420 min. The material prepared in this manner was the subject of further tests.
- Variant II: Cooled in water, the supersaturated samples were cold-rolled with 50% reduction (3.0 → 1.5 mm). The aging process was carried out using the same parameters as in Variant I.

The investigation methodology employed in the studies involves specific testing methods. The Vickers hardness measurement was carried out using a tester with a diamond indenter on the HV0.5 hardness scale (Hardness Tester Vickers/Knoop/Brinell Falcon 450, INNOVATEST Europe BV, Maastricht, Holand) Measurements were performed in line with based the ASTM E92 standard. Based on the values thus obtained, an average of 10 measurements was calculated for each sample.

The issue of electrical conductivity measurement errors was solved by introducing a mechanism for compensating the coil’s distance from the surface of the tested element. This is the widely known Förster mechanism. In general, the electrical conductivity of the alloyed copper samples was measured with reference to [45], and the electrical conductivity values obtained from 10 measurements were averaged for each sample.

The results of the hardness (HV) and electrical conductivity [MS/m] tests of the Cu-4Ti alloy following solution heat treatment and aging (Variant I) as well as after solution heat treatment–cold rolling (Z = 50%) then followed by aging are provided in Table 2.

### 2.2. Mathematical Modeling

Curve fitting (CF) is an efficient technique for mathematical modeling using experimental data, making it possible to effectively analyze multifactorial correlations. The resultant curves, which use various curve fitting strategies, must satisfy smoothness, precision, and physical feasibility constraints. In the case of the study in question, the MATLAB Curve Fitting (The MathWorks Inc., Natick, MA, USA) toolbox was used to perform curve fitting while considering a multifactorial and complex dataset. Upon initial testing, polynomial fitting appeared to be the best solution to provide the optimal representation of the curves.

Statistical data evaluation was conducted for the *Undeformed and Aged* and *Deformed and Aged* conditions in terms of electrical conductivity and hardness, as listed in Table 2. For the *Undeformed and Aged* conditions, the average electrical conductivity is 6.16 MS/m, while the standard deviation for this measurement is 1.45 MS/m. The 95% confidence for electrical conductivity ranges from 5.55 to 6.77 MS/m. For the *Undeformed and Aged* conditions, the average hardness is 206.10 HV5, while the standard deviation for this measurement value is 39.48 HV5, and the 95% confidence ranges from 189.42 to 222.77 HV5. For the *Deformed and Aged* conditions, the average electrical conductivity is 6.83 MS/m, while the standard deviation for this measurement is 1.95 MS/m, and the 95% confidence ranges from 5.91 to 7.74 MS/m. The average hardness under the *Deformed and Aged* conditions is 243.90 HV5, while the standard deviation for this measurement is 49.84 HV5, and the 95% confidence ranges from 220.57 to 267.23 HV5. These statistical investigations, based on the data listed in Table 2, enhance the robustness of the given data by including the necessary parameters of the statistics, thus enabling better data interpretation.

The MATLAB CF function can perform strong nonlinear 3D space regression using two parameters for the input and one for the output in order to effectively capture paired relationships. These were modeled by means of polynomial functions to obtain an inclusive model. Striving for efficient approximation of the experimental data with the minimum error, 3 and 5 were taken to obtain the polynomial order for axes x and y, respectively. Polynomial degrees were selected based on their fit to such statistical parameters as the sum of squares due to error (SSE), R-square, adjusted R-square, and root mean square error (RMSE). The setup yielded efficient polynomial models of regression (Equations (1)–(4)), which were derived using the experimental data provided in Table 2. The models fit the predicted behavior and are computationally efficient. Although the least effective coefficients in the equations are used in metaheuristic algorithms, they are omitted and presented below.

f_hardness_undef = −5495 + 32.38x + 306y −0.06172x^2^ + −1.788 xy + −3.75y^2^ 0.003511x^2^y + 0.02158xy^2^ + 0.001509y^3^ (1)

This polynomial function is between two variables, y (time) and x (temperature), and coefficients for all the terms representing hardness (f_hardness_nondef). The function is made up of interactions between y and x in linear, quadratic, cubic, and quartic forms, where coefficients define how much to contribute to f_hardness_nondef.

f_cond_undef = −85.45 + 0.5189x −2.675y + −0.00102x^2^ + 0.01525xy + 0.0337y^2^ + 6.715 ×10^−7^x^3^ −0.0002333xy^2^ + 0.0001259y^3^ (2)

The equation is used for a two-variable polynomial function to define conductivity (f_cond_nondef). Similarly to the function for hardness, in this function, one has added linear and higher-order interactions, such as quadratic, cubic, and quartic, between y and xx. The coefficients given for the foregoing define their individual contributions to total conductivity.

f_hardness_def = 9493 −54.67x −429.1y + 0.1061x^2^ + 2.494xy + 0.8578 y^2^ −0.004592x^2^y −0.00674 xy^2^ + 0.006644y^3^ (3)

The polynomial function assigns two parameters, y and x, to define f_hardness_def. The function consists of interactions between y and x, including linear, quadratic, cubic, and quartic, while the coefficients define the individual contributions of each to deformation hardness.

f_cond_def = 511.4 −2.985x −32.14y + 0.005801x^2^ + 0.1903xy + 0.2121y^2^ −0.0003727x^2^y −0.001278xy^2^ (4)

Deformation conductivity, f_cond_def, is described using a two-variable polynomial function, featuring linear, quadratic, cubic, and quartic components, where the coefficients define the relative contributions of individual interactions to deformation conductivity.

An important aspect to the discussion on whether or not a regression model is satisfactory is how well the values predicted fit the data observed. These may be measured using several parameters. In this study, MATLAB’s Curve Fitting application was used to calculate the RMSE, SSE, R-squared and adjusted R-squared values to benchmark the model’s performance. The measurement of R-squared (R^2^) expresses both the model’s fit and variance in the data observed, obtained by means of the model. An R^2^ approaching 1 stands for an excellent fit and reflects a strong association between the values observed and predicted. The SSE values calculated for the values for hardness and conductivity are 452 and 0.99, respectively. Considering that the hardness ranges at 137–300 and the conductivity ranges at 3–9.4, such low SSE values indicate a strong fit for both datasets. The R^2^ values obtained for hardness and for conductivity are 0.98 and 0.97, respectively, indicating an excellent fit between the experimental data and the polynomial model. Furthermore, the corrected R^2^ values for hardness and for conductivity are 0.97 and 0.98, respectively, giving evidence to the model’s robustness. Moreover, the RMSE values were calculated at 6.7 for hardness and at 0.27 for conductivity. Such low RMSE values provide evidence for the accuracy of curve fitting and indicate the strength of the polynomial approach in capturing developmental patterns in data. Therefore, the values obtained for the undeformed as well as for the deformed and aged data, as shown in Table 2, were found to ensure perfect modeling in terms of R^2^, corrected R^2^, SSE, and RMSE values.

**Figure 1:**
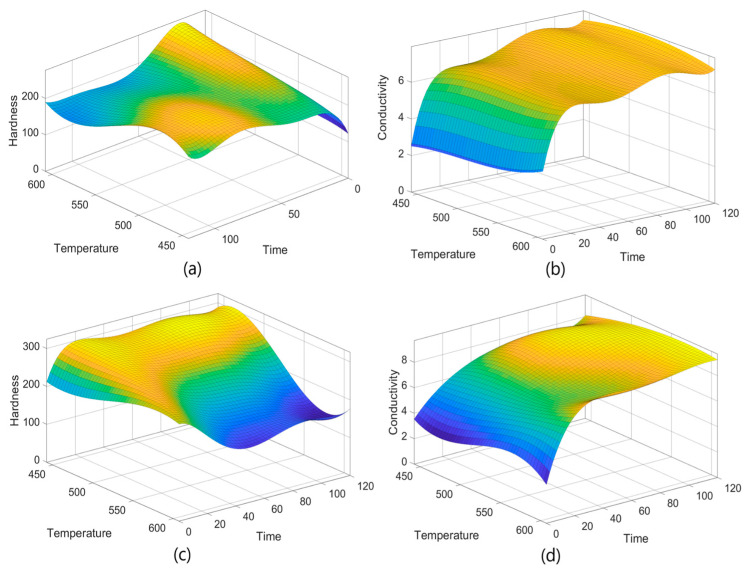
Three-dimensional graphs representing the *Undeformed and Aged* case (**a**,**b**) and 3D graphs representing the *Deformed and Aged* case (**c**,**d**) obtained using the polynomials given in Equations (1)–(4).

Figure 1 depicts the correlations between the independent parameters of aging time (x-axis) and temperature (y-axis) and dependent parameters such as hardness and conductivity. The plots have been described using nonlinear polynomial expressions (Equations (1)–(4)) developed using the MATLAB Curve Fitting tool. Figure 1a,b show the distributions of hardness and the conductivity for initial and aged materials, while Figure 1c,d illustrate the same parameters for plastic deformed and aged materials. In the absence of deformation and aging, time and temperature have a significant influence on hardness (Figure 1a). The increase in hardness, especially under elevated temperature and prolonged aging conditions, is attributable to microstructural modifications in the material. In the conductivity (Figure 1b) graph, the nonlinear interaction between temperature and time is remarkable. The conductivity decrease at high temperatures and long aging times clearly reveals the effect of the aging process on the electrical properties of the material. In the deformed and aged condition, the hardness (Figure 1c) shows a significant increase because of deformation. This sharp hardness increase, especially under prolonged and high-temperature aging conditions, is attributed to deformation-induced strengthening via treatment by aging. Conductivity (Figure 1d) is observed to have a mixed influence on deformation. An increase in conductivity is observed under low-temperature and short-term aging conditions, while under prolonged and high-temperature aging conditions, conductivity declines. This is related to the effects of deformation and aging on the microstructure of the material. In conclusion, both the graphs thus obtained and the related polynomial models provide a powerful tool for understanding the effects of deformation and aging processes on the mechanical and electrical properties of the material. The high R^2^ and adjusted R^2^ values and the low RMSE and SSE values, indicating modeling accuracy, prove that the chosen polynomial approach is clearly suitable and conforms with the experimental data.

**Figure 2:**
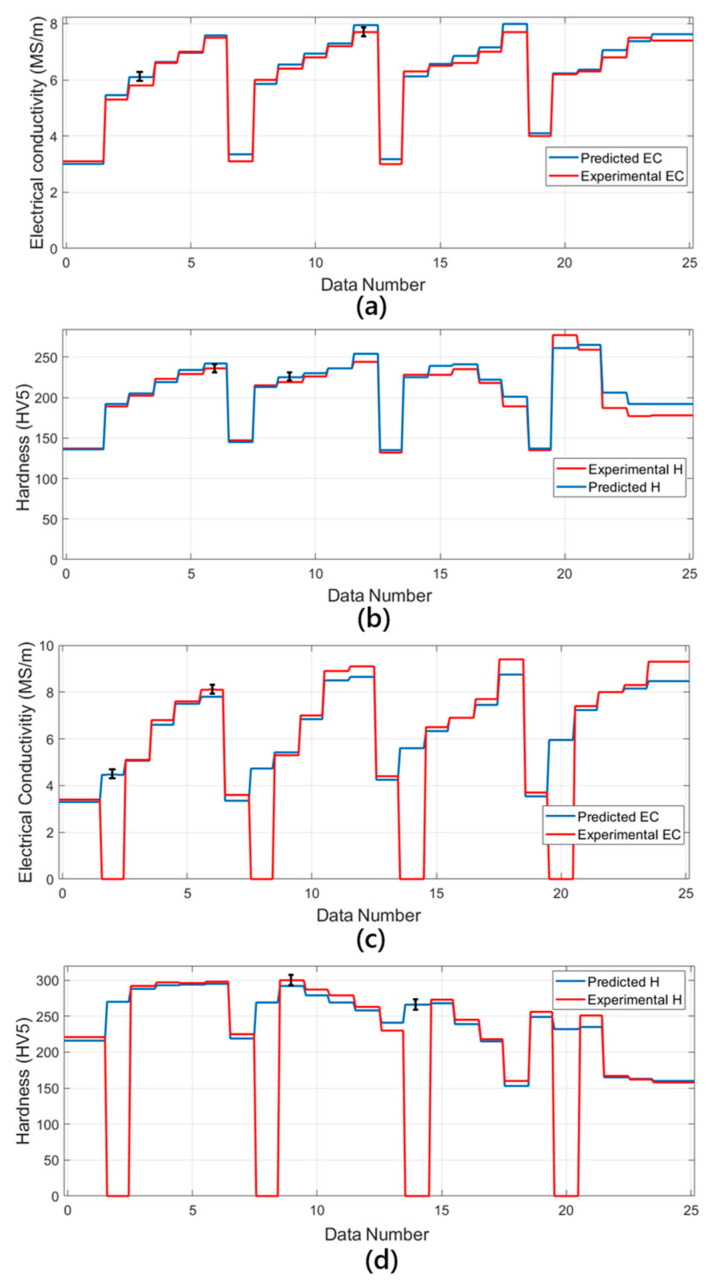
Comparison of the experimental and predicted values for electrical conductivity (EC) and hardness (H) in different process conditions. (**a**,**b**) represent the *Undeformed and Aged* process, while (**c**,**d**) correspond to the *Deformed and Aged* process.

Figure 2 provides a comparison of the experimental and predicted data. The subplots in Figure 2a,b show the electrical conductivity (EC) and hardness (H) data obtained for the *Undeformed and Aged* process. The graphs clearly evidence that the nonlinear regression model provides a high degree of agreement with the experimental data. The model has successfully captured the general trends in the data and largely overlaps with the experimental data, except for small deviations. The Figure 2c,d subplots show the experimental and predicted data for the deformed and aged process. What one can observe in these graphs is that the experimental data drop to zero at certain points. This indicates that there are no data points at the points of interest. However, the nonlinear regression model has predicted a value in between these gaps and showed a reasonable trend by maintaining continuity. This illustrates that the model can accurately capture the overall trends when there are no data points and make reasonable predictions in data gaps. Thus, the nonlinear regression model used seems to provide reliable predictions for both *Undeformed and Aged* as well as deformed and aged processes. The model stands out as an effective forecasting tool, especially by making reasonable predictions for missing data points.

## 3. Implementation and Results

### 3.1. Characterization of Materials

Among copper alloys, beryllium copper is characterized as the one with the best strength properties, high electrical conductivity, as well as resistance to corrosion and abrasion [46,47,48]. One of the most important advantages of these alloys is the lack of tendency to spark. However, copper alloys with the addition of beryllium are very toxic, especially during heat treatment and plastic processing [49,50,51]. Similarly, cadmium copper (CuCd), which is commonly used for the production of electric traction wires or railway traction network systems, is toxic as well. For this reason, the use of the CuBe and CuCd alloys in the EU countries is associated with serious restrictions [52].

The search for alternative substitutes for the CuBe alloys has brought various outcomes, including the research on CuTi- and CuNiSi-alloyed copper. In comparison to the CuBe-alloyed copper, they are characterized by similar electrical properties and comparable mechanical properties [47,50,53]. The CuTi alloys also show no tendency to spark. Therefore, taking into account the mechanical properties of the CuTi alloy, one can assume that it should be a potential substitute for the toxic types of alloyed copper such as CuBe or CuCd.

The conclusion arising from a comparison of the mechanical properties and electrical conductivity of beryllium copper with industrial grades of alloyed copper is that the industrial alloys offer better properties in a wider temperature range [47].

CuTi alloy copper grades meet the requirements for resistance to stress relaxation at high temperatures. As a result, they are used for the production of high-performance flexible elements used in the electronics industry as well as high-performance conductive flexible elements for the aerospace and microelectronics industries [54]. They are also used for the production of switches, connectors, and other electronic devices.

Computational analyses and experimental results have shown that the combination of conventional heat treatment and cold rolling before aging can lead to significant improvements in microstructural features, thus improving the alloy’s overall performance. The results of this study provide a basis for optimizing critical properties such as hardness and electrical conductivity, which can be easily adapted to industrial applications. The metaheuristic algorithms employed while studying the CuNi2Si-alloyed copper, including GA, PSO, and SPBO, accurately predicted the optimal aging parameters, effectively resolving the complex problem of interdependence between temperature, heat treatment time, and mechanical deformation. SPBO can be performed with high efficiency, minimal computational cost, and high prediction accuracy [41]. The polynomial models implemented in the optimization environment confirmed the prediction values with a high R-squared values of the coefficient of determination at 0.98 and 0.96 for the confidence level [41].

The impact of these results is straightforward, and they can be applied in industries such as aerospace, electrical engineering, and high-tech manufacturing. By synergistically combining experimental methodologies with computational approaches, metaheuristics provide a solid basis for predicting the best processing conditions with significant savings in costs and experimental time [41].

Having reviewed the literature on the subject [55,56,57,58] released to date, one can conclude that the copper alloys containing 2–6% Ti, usually referred to as titanium copper, are the most promising variety as they can be utilized primarily in the energy and electronics industries as well as for the production of mine rescue and anti-terrorist equipment [59]. This results from the following facts:

- The most effective way to increase strength properties is to apply precipitation hardening combined with strain hardening. For this reason, studies are being conducted on the effect of combined heat treatment with cold rolling between the supersaturation and aging operations and after aging [16,18,60]. It has been found that the occurrence of spinodal transformation is crucial for strength properties [61].
- The improvement of strength properties and electrical conductivity can be controlled by changing the precipitation and recrystallization kinetics. For this reason, various processing variants are applied, comprising a combination of a sequence of heat treatment and cold rolling operations [5], inter-operational rolling after a bath in liquid nitrogen [62], aging in a hydrogen atmosphere [63,64], as well as heat treatment and hot plastic deformation [65,66,67]. Some other viable options include introducing another alloying additive to titanium copper [68,69,70] or producing alloy copper by methods other than the conventional ones [71], resulting in a significant improvement in the material properties.

What has been found in the studies conducted to date is that solutionization is a critical and decisive process that determines the final microstructure and properties of CuTi alloys. Therefore, it is important to precisely determine the effect of solutionization conditions on the microstructure before the next stage of processing, which is ageing. The above data show that the supercooling process and, in particular, the pre-cooling time have a significant impact on improving the properties. An appropriately long soaking time should ensure complete dissolution of the alloying element in the matrix. In the CuTi alloy copper, the post-solutionization hardness is affected by the titanium content [62].

Therefore, in order to find the optimal soaking time before cooling (supersaturation), samples were soaked at 900 °C for 5, 15, 30, 60, and 120 min. Based on the results of the electrical conductivity and HV tests, it was determined that the optimal soaking time was 60 min.

**Figure 3:**
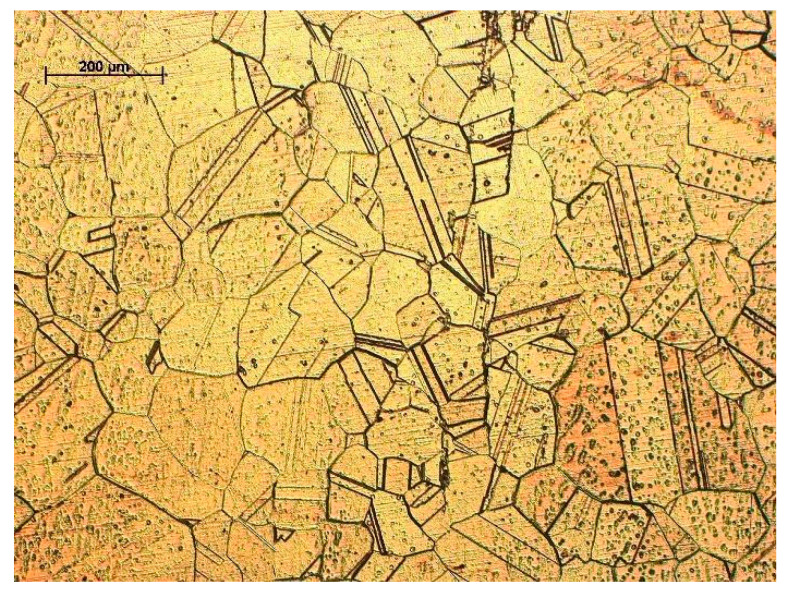
Microstructure of the industrial Cu-4Ti alloy supersaturated at 900 °C for 1 h and cooled in water.

Depending on the titanium content, titanium copper is supersaturated in the temperature range of 700–950 °C for up to 7 h and then aged at 400–600 °C for 1 to 16 h [52,72]. For titanium copper with a Ti content above 2%, the supersaturation time ranges from 30 to 120 min. In the case of titanium copper with a Ti concentration below 2%, extending the supersaturation time in the range of 1–4 h does not significantly affect the change in hardness after supersaturation, but the homogeneity of the supersaturated Ti solution in the matrix increases [73]. The Cu-4Ti alloy after solution annealing is characterized by a coarse-grained microstructure with a large number of twin boundaries [63] and fine undissolved Ti particles (Figure 3).

**Figure 4:**
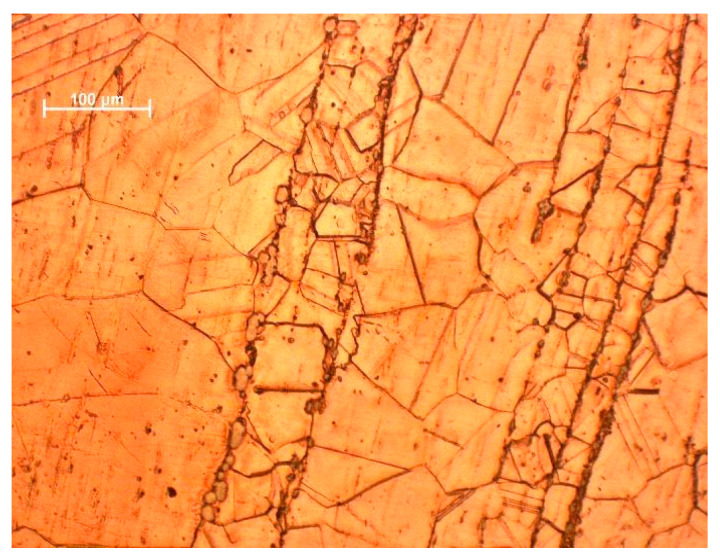
Microstructure of the Cu-4Ti alloy following solution heat treatment and cold rolling (Z = 50%).

Microstructure studies have confirmed the inhomogeneity of deformation during cold rolling. This is the consequence of the uneven dissolution of titanium in the copper matrix. The characteristic deformation bands are those in which the grain diameter is significantly smaller than in the other areas (Figure 4) [73].

In the microstructure of the supersaturated and cold-rolled alloy (50% reduction), numerous deformation bands were found, whose average grain diameter was several hundred percent smaller than that of the grains outside the bands. This is the effect of the uneven dissolution of titanium in the matrix, which resulted in different Ti concentrations in neighboring grains.

**Figure 5:**
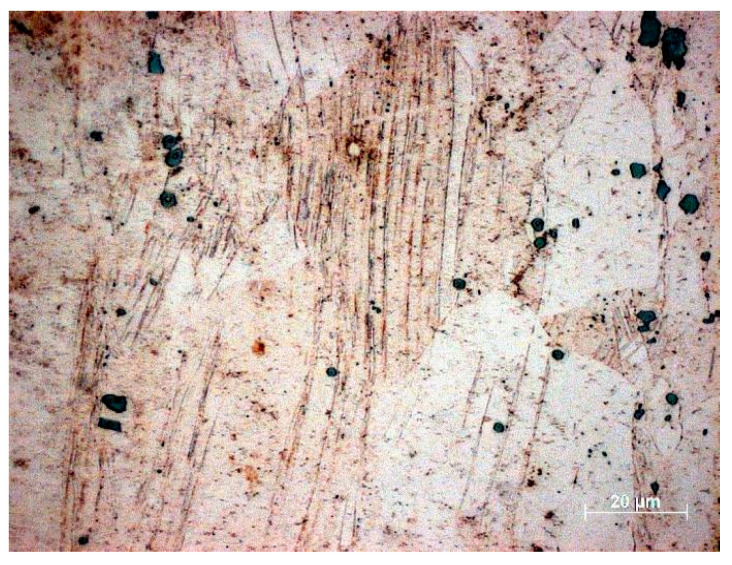
Microstructure of the Cu-4Ti alloy following solution annealing and aging at 600 °C for 60 min.

Following solution heat treatment and subsequent aging at 600 °C for 60 min, no grains with an average diameter greater than 50 μm were found in the microstructure (Figure 4). Grains with an average diameter ranging at 20–50 μm and a shape close to spherical dominate. No annealing twins were found, but lamellar precipitates were visible in the grains (Figure 5).

**Figure 6:**
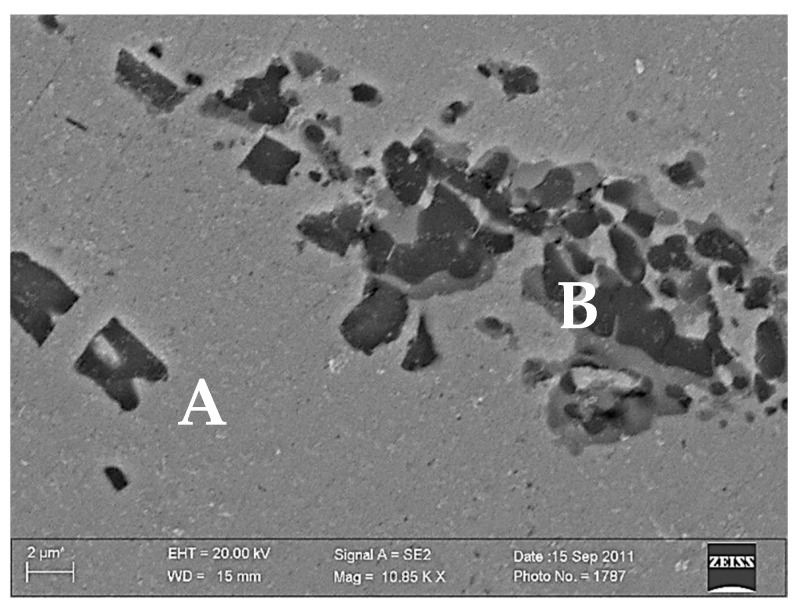
Microstructure of the Cu-4Ti alloy following solution heat treatment with chemical composition analysis in micro-areas (EDS); area A (Ti 2.6%; Cu 97.4%) and area B (Ti 89.7%; Cu 10.3%); SEM.

Microstructure analysis using a scanning electron microscope (SEM) together with micro-domain chemical analysis (EDS) have revealed that the matrix consists of copper with titanium dissolved in it and a few undissolved Ti particles (Figure 5). The grain boundaries of the supersaturated solid solution are free from precipitates, and a subgrain structure is visible at the grain boundary (Figure 6).

**Figure 7:**
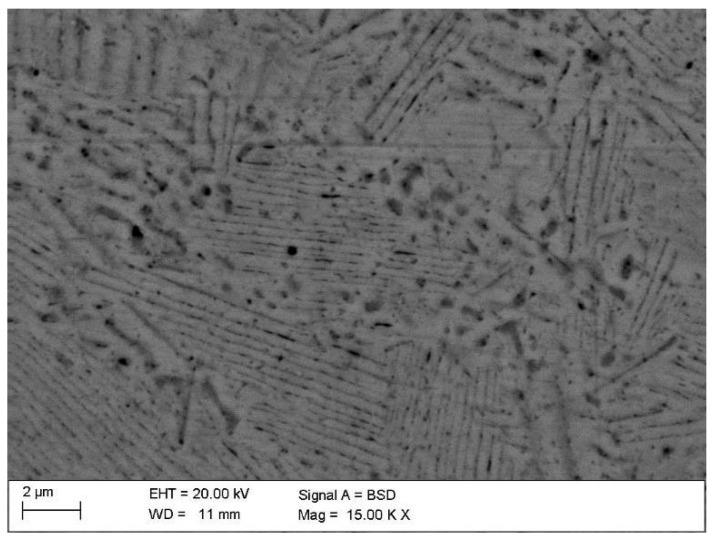
Microstructure of the Cu-4Ti alloy supersaturated and aged at 600 °C for 60 min with visible lamellar precipitations.

**Figure 8:**
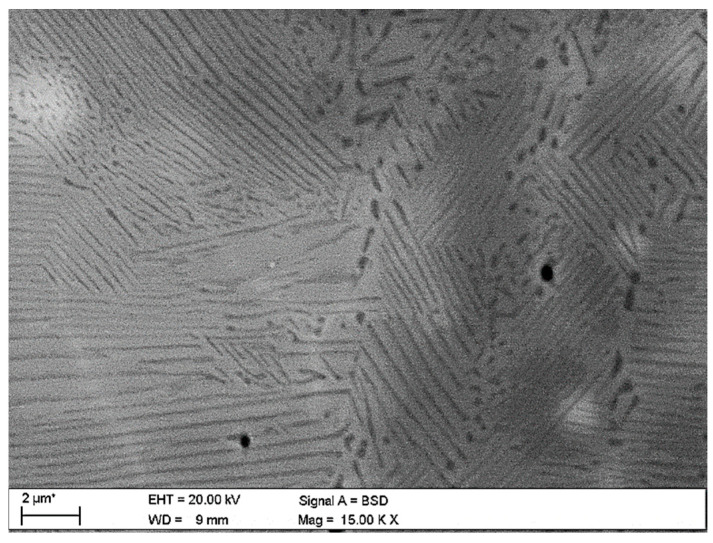
Microstructure of the Cu-4Ti alloy supersaturated, cold-rolled, and aged at 550 °C for 420 min with visible lamellar precipitations.

During aging, as a result of discontinuous transformation, the Cu_3_Ti phase is precipitated in the form of plates arranged alternately with plates of the solid solution, occurring both in the non-plastically deformed alloy (Figure 7) and in the cold-rolled alloy (Figure 8). As a result of spinodal transformation, coherent precipitates were formed, periodically distributed in the matrix. The chemical composition of the equilibrium phase and the microstructure cannot be explicitly determined. Based on the chemical analysis of the areas with different image contrasts, two phases can be distinguished: the disordered β′ phase and the ordered β phase.

**Figure 9:**
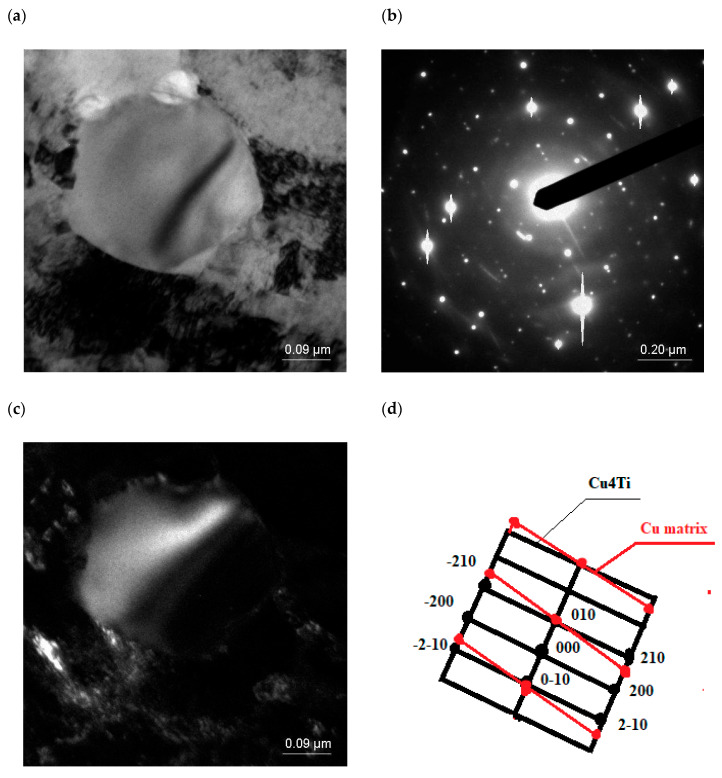
Structure of the Cu-4Ti alloy supersaturated at 900 °C: (**a**) bright field image; (**b**) diffraction pattern from the area as in (**a**); (**c**) dark field image from reflection 6 of the Cu_4_Ti phase crystallizing in the tetragonal lattice (space group I4/mmm); (**d**) solution of the diffraction pattern presented in (**b**).

Following examinations using a transmission electron microscope (TEM), it was found that the microstructure of the supersaturated Cu-4Ti alloy in the Cu(Ti) matrix contained recrystallized subgrains with visible annealing twins (marked with an arrow) inside (Figure 9).

**Figure 10:**
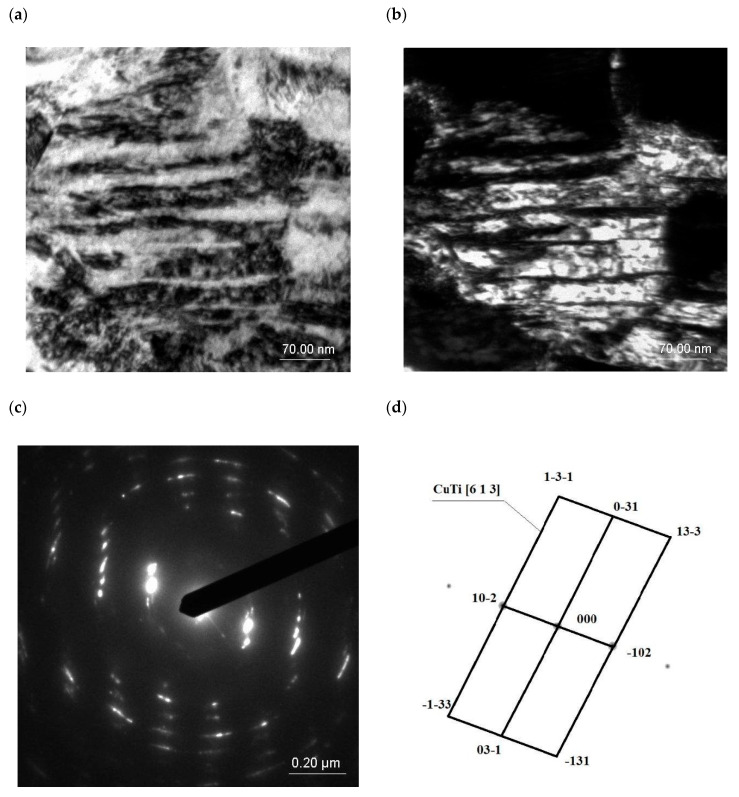
Microstructure of the Cu-4Ti alloy: (**a**) bright field image; (**b**) diffraction pattern from the area shown in (**a**); (**d**) solution of the diffraction pattern from (**c**) with the CuTi phase in the [6 1 3] zone axis; (**c**) dark field image from the 113 reflection of the Cu matrix with a cubic lattice (space group Fm-3m).

After solutionizing from a temperature of 900 °C and subsequent cold rolling with a reduction degree of Z = 50%, deformation twins were revealed, which were also visible in the dark field image (Figure 10). The diffraction solution also made it possible to identify the second phase, namely Cu_3_Ti. The presence of the Cu_3_Ti phase after solutionizing was also confirmed in the X-ray phase analysis.

**Figure 11:**
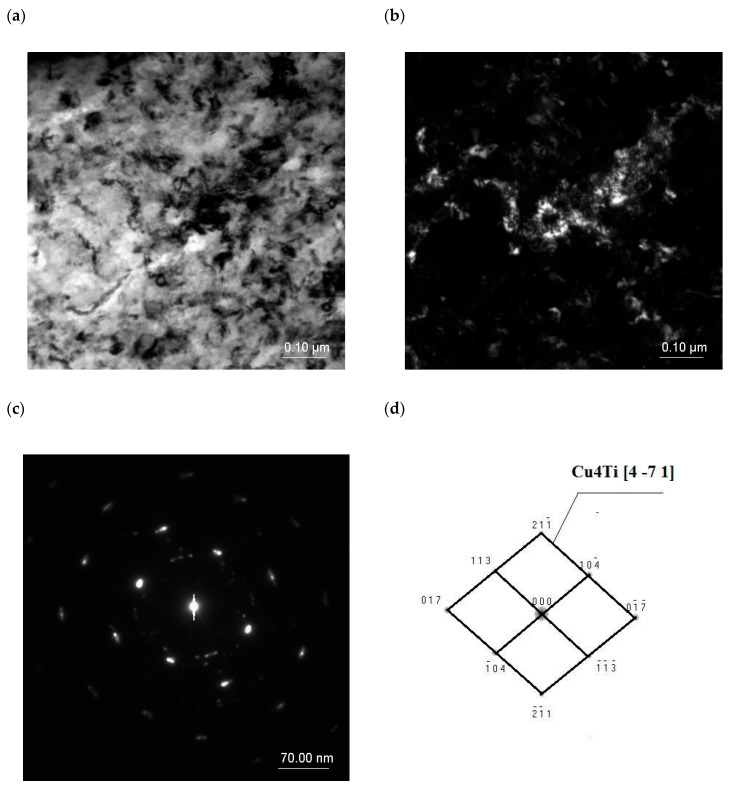
Structure of the Cu-4Ti alloy after aging at 550 °C for 30 min: (**a**) bright field image; (**b**) diffraction pattern from the area as in (**a**); (**d**) solution of the diffraction pattern from (**b**); (**c**) dark field image from the -1 1 3 reflection of the Cu_4_Ti phase crystallizing in the orthorhombic lattice (space group Pnma).

α’-Cu_4_Ti particles were found to be present in the microstructure of the supersaturated alloy, rolled and aged at 550 °C for 30 min (Figure 11), but extending the aging time to 420 min caused dissolution of the precipitated particles. Similar results were reported to have been obtained in [74].

Table 3 contains the results of the hardness (HV) and electrical conductivity [MS/m] tests of the Cu-4Ti alloy after solutionizing.

### 3.2. Optimization Performance of Metaheuristic Algorithms

Metaheuristic optimization algorithms have been extensively applied to solve scientific and engineering problems, making it possible to effectively search through large search spaces and avoid local optima. Solutions such as: Artificial Hummingbird Algorithm (AHA), Ant Lion Optimizer (ALO), Atom Search Optimization (ASO), Equilibrium Optimizer (EO), Flow Direction Algorithm (FDA), Moth-Flame Optimization (MFO), Marine Predator Algorithm (MPA), Multi-Verse Optimizer (MVO), Particle Swarm Optimization (PSO), and Salp Swarm Algorithm (SSA) have been broadly utilized on account of their capacity to effectively optimize nonlinear, multi-dimensional, and constrained problems. The potential of such algorithms to provide successful outputs depends to a large extent on the accurate specification of the fitness function, which is an integral factor guiding the search towards the optimal solution. However, due to some intricacies and limitations of the problem at hand, in some scenarios, the ideal solution may never be reached. Such limitations highlight how important it is to design the fitness function carefully in order to provide robust and reliable optimal outputs. In this study, the fitness function used to optimize the heat treatment process parameters is defined in Equation (5), representing a systematic means to assess and optimize process parameters. The methodology proves efficient when addressing real-life constraints in optimization and effectively facilitates algorithmic convergence towards an effective and realistic solution.

fitness=A *x* |f_(hardness_des)-f_hardness|+ B *x* |f_(conductuvity_des)-f_conductuvity| + C × f_energy (5)

In this case, f_hardness_des and f_conductivity_des are to-be-achieved output parameters, while f_hardness and f_conductivity are the polynomial expressions in Equations (1)–(4). In this case, f_energy is the total energy expenditure in the heat treatment operation. The parameter is specified in terms of the power for the curing furnace and the time for the heat treatment operation. The addition of energy expenditure in the fitness function causes the optimizing algorithm to perform optimization for the values of required hardness and conductivity using minimum energy, hence the optimization for enhanced process efficiency.

The weighting coefficients of B, A, and C are included to modulate the relative impact of each term in the fitness function. The coefficients allow flexibility in prioritizing different goals of optimization, steering the search process of the metaheuristic algorithm. The capability of metaheuristic algorithms to modify their strategies for exploration and exploitation in response to dominating terms in the objective function is well established. If any given factor is weighted more, the algorithm is predisposed to perform optimization in favor of such a factor, converging rapidly to an optimal solution in the given subdomain of the search space.

In this case, the assumed coefficients were A = 10, B = 10, and C = 1. These coefficients favor optimizing to reach the necessary values for hardness and conductivity, while minimizing is secondary. The coefficients can be adjusted to guide the optimizing process in different directions, akin to the roulette wheel system, where different tilting angles guide the choice probability towards different optimizing objectives.

The hyperparameters of the metaheuristic algorithms shown in Table 4 were defined by using the ranges of values and optimal settings proposed in the literature to improve the performance of individual algorithms. They were selected by referring to the parameter settings most frequently employed according to basic operation mechanisms of individual algorithms, and achievements in various applications were taken into account.

**Figure 12:**
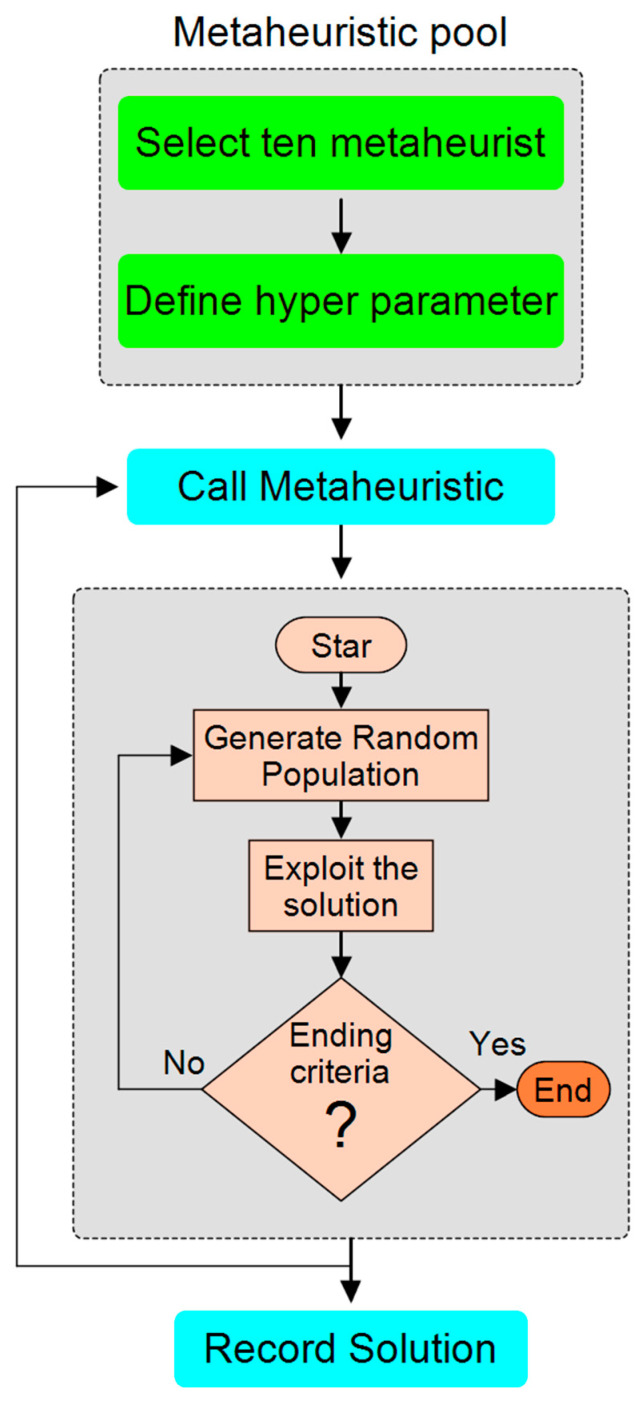
Workflow of the metaheuristic selection and optimization process.

Figure 12 illustrates the workflow procedure for selecting and executing metaheuristic algorithms. The pool of metaheuristics is first developed, and ten algorithms are selected. Hyperparameters are defined to optimize the performance of the chosen algorithms. The selected metaheuristic algorithm is called, and the procedure for optimization is initiated, which begins by generating an initial population, and afterwards, potential solution exploitation is performed. The loop is traversed by the algorithm until termination is achieved, whereupon the optimization is finalized; otherwise, iterative running is resumed. The solution is recorded in the last operation. The systematic procedure depicted in Figure 12 ensures systematic and efficient testing of the given metaheuristic algorithms for optimization problem solving.

**Figure 13:**
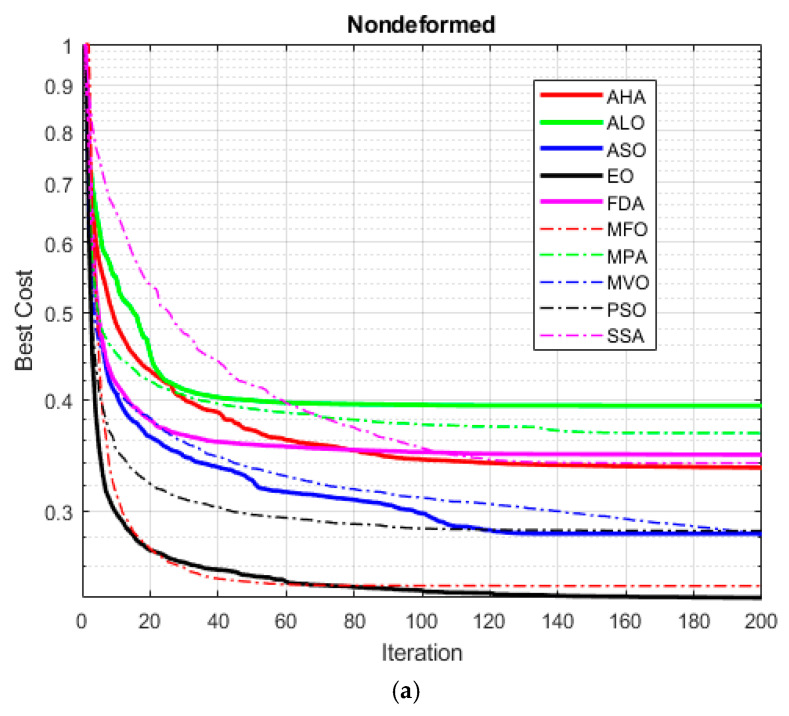

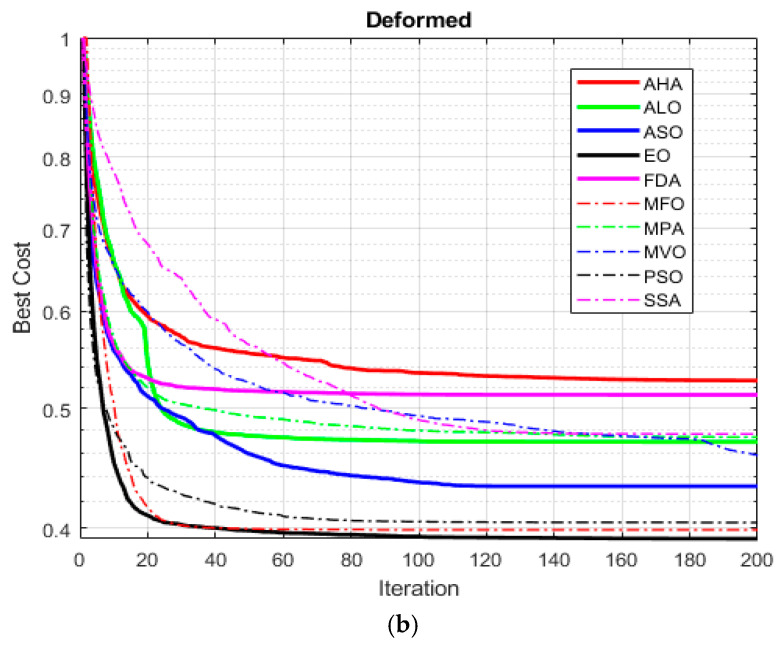
Comparison of the best cost values of different metaheuristics by iteration: (**a**) undeformed process and (**b**) deformed process.

Figure 13a illustrates a comparative analysis of the converging behaviors of AHA, ALO, ASO, EO, FDA, MFO, MPA, MVO, PSO, and SSA under the nondeforming process conditions. The assessment is made based on the optimal cost values as per the iterations. The behavior of the Equilibrium Optimizer (EO) and Moth Flame Optimization (MFO) is found to be superior to the others when it comes to reaching the minimum cost values on account of their rapidly converging behavior. The Flow Direction Algorithm (FDA), Atom Search Optimization (ASO), and Particle Swarm Optimization (PSO) show good behavior after a sufficient number of iterations, but their converging behavior is slow in comparison to EO and MFO. The Marine Predator Algorithm (MPA) converges in the middle, whereas Ant Lion Optimization (ALO), the Salp Swarm Algorithm (SSA), and Multi-Verse Optimization (MVO) show no noticeable advancements after completing enough iterations, and their behavior is stuck in the local minima.

Figure 13b provides the convergence plot of the deformed process. The EO and MFO algorithms differentiate after reaching their minimum cost values due to their capability to converge rapidly. The FDA, ASO, PSO, and MPA also exhibit good convergence, but after a certain number of iterations, their rate of improvement becomes inferior to EO and MFO. Moreover, while ALO and SSA do show an initial significant cost decrement, their convergence is stuck in future iterations. The MVO algorithm only progresses to a certain limit, having problems converging to low-cost values, whereas the AHA yields improvements in convergence but stabilizes to larger-cost values compared to other algorithms.

To provide a valid judgment of the strengths and weaknesses of the optimization methods used in this research and despite the balanced exploration and exploitation mechanisms of EO, as well as its decent performance in big search spaces, it shows the tendency to fall into the local minima and is parameter-dependent. Although MFO has the property of exhaustive search and is efficient at attaining the global solution with its chaotic mechanism, the convergence speed may be affected because there is no exploitation in the later stages of the optimization process. Even though FDA is efficient in dynamic systems owing to its flow direction-based optimization capability, its convergence speed may be lower in comparison to other algorithms when tackling very complex problems. Although ASO shows strong performance in terms of the exploration and exploitation balance thanks to its pull and push mechanisms, its efficiency may decrease due to the high computational costs associated with large problems. PSO has the advantage of fast convergence and fewer parameters but may not perform enough exploration in some cases as it can get stuck in local minima and features less randomness. Even though MPA is suitable for dynamic optimization problems, given the predator–prey mechanism, it may reveal instability in convergence and may also pose the risk of premature convergence. Although ALO offers effective exploration with its random walk and trap mechanisms, its convergence speed may remain low in cases of high-dimensional problems. While SSA offers the advantage of fast convergence and low computational costs, it may tend to converge early on large datasets. Although MVO offers a wide exploration area with black hole and white hole mechanisms, it entails the risk of getting stuck in local optima. Even though AHA exhibits strong exploration capabilities thanks to its nature-based motion dynamics, it may show slow convergence in some problems. However, the success of metaheuristic algorithms depends not only on the algorithm structure but may also vary according to parameters such as the properties of the function to which they are applied, the width of the search space, the problem size, and the complexity of the solution surface. Therefore, the effectiveness of any optimization algorithm should be evaluated in the context of a specific problem set, and it should not be assumed that a single algorithm will provide the best solution for all problems. For this reason, the presented study provides a comprehensive evaluation of the advantages and limitations of the algorithms used in optimization processes.

Table 5 summarizes the outcomes of the ten different metaheuristic algorithms applied to optimize the prediction of the optimal aging time and optimal aging temperature in a heat treatment operation. The left column of the table lists 15 different data codes, and for every code, there is a desired EC and H. The objective of the optimizing procedure is to recognize the optimal aging time and optimal aging temperature to obtain the desired EC and H. The optimizing procedure is performed using the fitness function in Equation (5) and takes into consideration both the deviation in the EC and H values and the amount of energy expended in the procedure. The metaheuristic algorithms applied in an iterative procedure, including AHA, ALO, ASO, EO, FDA, MFO, MPA, MVO, PSO, and SSA, adjusted the procedure parameters to optimize the fitness function in order to approach the optimal solution. Data D1–D4 and D9–D12 among those provided in Table 5 have taken the values from Table 2, containing the experimental data. The rationale behind including such data is to verify whether the regression developed, the optimizing procedures employed, and the hyperparameters chosen are sufficient. Experimental data and optimizing data are compared to verify whether the computational procedure employed is efficient. The coincidence between the predicted and experimental data shows whether the optimizing procedures employed are reliable and accurate.

Using the resultant values, variations are found in their prediction for the optimal parameters of aging against different metaheuristic algorithms. These variations are caused by their different search mechanisms, their converging speeds, and their trade-offs between their exploitations and their explorations. For instance, while AHA, EO, FDA, and SSA produced virtually identical values based on different datasets (i.e., D1, D4, D5, D6, and D9), indicating strong consistency in their optimizing procedure, algorithms such as PSO and MVO, on the other hand, showed significant disparities in the optimal parameters for the optimal values in datasets such as D8 and D10, where their predicted values for their aging time and temperature differed considerably. These disparities may lead to their different converging behaviors and local minimum sensitivity. Moreover, in dataset D8, while most of them converged to 478.1 °C for their aging temperature and 120 min for their aging time, several of them, including MVO, PSO, and SSA, produced significantly dissimilar optimal values for their respective aging conditions (600 °C and 20.2 min). These values indicate that, while some algorithms may have prioritized minimizing their intake of energy, others have prioritized optimizing only their resultant materials’ values.

**Figure 14:**
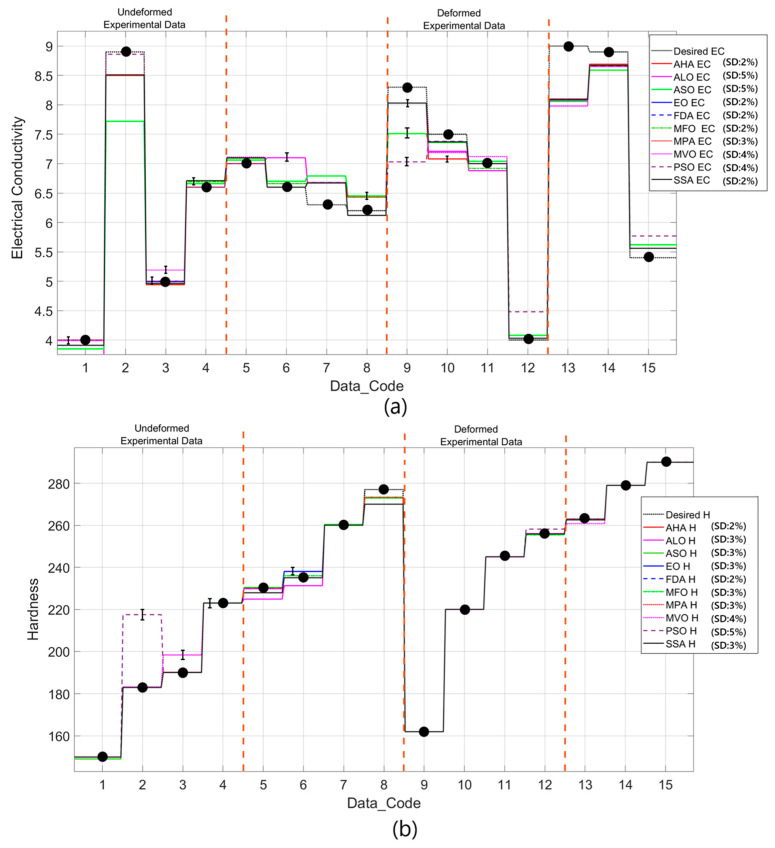
(**a**) Electrical conductivity and (**b**) hardness estimated using metaheuristic algorithms (SD: standard deviation).

Figure 14 depicts the predictions obtained by applying ten different metaheuristic algorithms for specific values of electrical conductivity (EC) and hardness (H) against dataset codes. The D1–D4 and D9–D12 data in Figure 14 have been directly taken from Table 2, while the remaining data were given arbitrarily. The data in Figure 14 which are represented by the black dots were assumed for the assessment of the prediction quality of the metaheuristic algorithms used. The discrepancy between the values predicted for EC and the desired experimental values, as shown in Figure 14a, is clear and demonstrates how different algorithms rank in performance. For certain data codes (e.g., D1, D4, D9, and D12), most algorithms closely approximate the desired EC values, indicating that they successfully converge to the experimental results. However, in cases of some algorithms, small deviations are observed, especially in the transition regions where conductivity changes abruptly. These deviations indicate differences in the capacity of each algorithm to adapt to abrupt changes in the response function. Algorithms such as EO, FDA, MPA, and AHA exhibit smoother approximation over the entire dataset, while others, such as ASO, ALO, and PSO, exhibit slight oscillations around the experimental values.

Similarly, Figure 14b depicts the metaheuristic predictions for hardness. The algorithms exhibit a relatively consistent trend in predicting hardness values, and there is a high degree of agreement between them. The alignment of the predicted hardness values with the black data points confirms the robustness of the optimization approach. At certain critical transition points (e.g., D4, D9, and D12), some algorithms show superior approximation capabilities, while others slightly underestimate or overestimate the desired values. MPA, FDA, and AHA seem to be more stable in their predictions, while MVO and PSO show small fluctuations. The deviations observed between the experimental data and the predicted values can be attributed to the differences between the search mechanisms, the convergence rates, and the exploration–utilization trade-offs of the metaheuristic algorithms. While some algorithms prioritize global exploration, others tend to exploit the local minima more aggressively, leading to differences in prediction accuracy. Furthermore, the fitness function type is also crucial in terms of how such optimization steps are guided. Given that the weight coefficients in the fitness function give priority to minimizing deviations in electrical conductivity and stiffness, such algorithms deliver an optimal compromise but result in minute variations in their resultant prediction. In general, Figure 14 demonstrates how efficient these metaheuristic algorithms are in approximating such sought-after material properties, despite different algorithms displaying better and worse converging behaviors.

**Figure 15:**
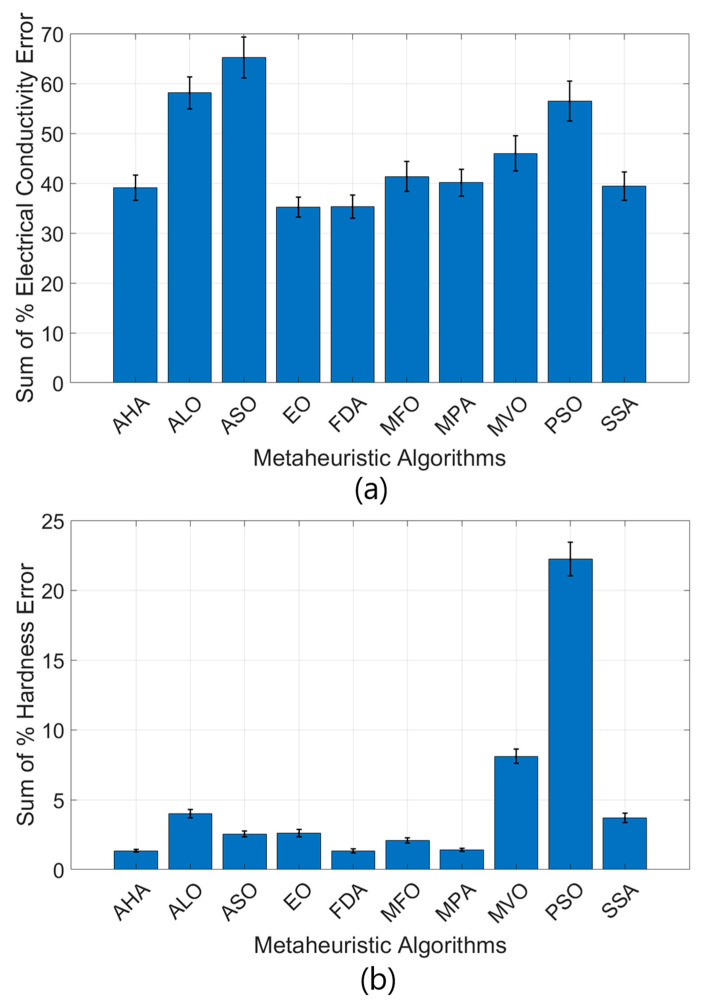
Comparison of metaheuristic algorithms based on the (**a**) % total conductivity error and the (**b**) % total hardness error. Lower error values indicate better performance, with EO and FDA showing the most consistent results.

In Figure 15, the performance of the metaheuristic algorithms is compared to the % total conductivity error and the % total hardness error. The rate of error is simply the total of 15 data point percentage values for the calculating error. The relatively lower values for the % total hardness error are derived from their larger numerical values, and hence their larger weight in the fitness function. As such, metaheuristic algorithms attach greater priority to minimizing the % total hardness error and therefore tend to exhibit lower error percentages for this factor.

Figure 15a clearly implies that, for the total conductivity error percentage, the FDA and EO algorithms achieved the minimum rate of error at 35.16% and 35.31%, respectively. Similarly, low error percentages were observed for AHA (39.13%) and SSA (39.42%). In contrast, in the case of the ASO algorithm, the rate of error was found to be 65.20%. Additionally, the PSO and ALO algorithms yielded comparative values of 56.42% and 58.14%, respectively. With reference to Figure 15b, in respect of the total percentage of the total hardness error, the minimum rate of error was found in the case of FDA (1.33%), followed by the AHA (1.34%) and MPA (1.40%). The highest rate of error occurred in the case of the PSO algorithm (22.25%), while MVO ranked second for the rate of error (8.11%). The remaining algorithms yielded values ranging between 2% and 4%. The increased weight-to-hardness ratio in the fitness function attracted more attention to the optimization of this factor, hence the minimized total rate of error for the % total hardness error.

An analysis of standard deviations illustrates how the PSO algorithm is found to display large variability in both % total conductivity error and % total hardness error, as their large error bars imply. The consequence is that PSO produces less consistent and less stable solutions. The EO and FDA algorithms, by contrast, are characterized by lower error values, and hence their smaller error bars, indicating better output consistency. Thus, EO and FDA can be ranked as the most efficient metaheuristic optimizing tools in terms of both % total conductivity error and % total hardness error.

This study comprised optimization of the hardness and electrical conductivity of the Cu-4Ti alloy, which depends on the aging temperature and time. The results obtained using metaheuristic algorithms revealed the performance levels of different algorithms in the optimization process. According to the data presented in Figure 15, FDA and AHA (Artificial Hummingbird Algorithm) delivered the most satisfactory results, while PSO and ASO exhibited the lowest performance. This can be explained by the nature of the algorithms used, their search mechanics, and their suitability to the problem structure. FDA is an optimization algorithm based on the principle by which liquid flow is directed in nature. Thanks to the balance of exploration and exploitation, it produced the best results by moving effectively in the solution space. The possible reasons for the superior performance of FDA can be explained as follows. The adaptive flow mechanism directs the search process in line with the problem space and prevents the algorithm from becoming stuck in the local minima. It ensured high accuracy in hardness and conductivity optimization by providing a more flexible movement capability in multi-dimensional solution spaces. All these advantages make FDA stand out as the most successful algorithm, with error rates of 35.16% in electrical conductivity optimization and 1.33% in hardness optimization. AHA was developed by taking inspiration from the feeding and migration behaviors of hummingbirds in nature. As shown in Figure 15, its performance was very similar to that of FDA, with error rates of 39.13% in electrical conductivity optimization and 1.34% in hardness optimization. The memory-based search mechanism that remembers past solutions in AHA provides more efficient solutions. The ability to dynamically switch between local and global search increases the ability to reach the optimum solution against multivariate engineering problems. The use of different search modes (feeding, exploration, and migration) provides more effective scanning of the solution space and a faster access to the optimum solution. These features enabled AHA to deliver high-accuracy results in the optimization of the Cu-4Ti alloy.

On the other hand, PSO is an algorithm based on the swarm intelligence principles applicable in nature. However, in this study, it exhibited low performance by showing the highest error rate (22.25%) in hardness optimization. The main reason for the failure of PSO may be the premature convergence problem. For multi-dimensional and opposing target functions (such as hardness and conductivity), PSO may not have sufficient discovery capacity. The ASO algorithm is founded on a physics-based atomic motion model. However, it proved to be the least successful algorithm with the highest error rate (65.20%) in electrical conductivity optimization. The reasons for the failure of ASO can be explained as follows: it was not flexible enough in very large solution spaces, especially for multivariate problems in materials science, and it did not provide a suitable optimization strategy. Additionally, in the search process, it tended to identify too many local solutions, which reduced the chance of reaching the global optimum.

The main reasons for the low performance of the Ant Lion Optimizer (ALO), Marine Predator Algorithm (MPA), Salp Swarm Algorithm (SSA), and Equilibrium Optimizer (EO) used in this study are the inability to sufficiently explore the problem space, difficulty in reaching the global optimum, and tendency to converge early. While ALO experienced the problem of becoming stuck in the local minima, MPA was insufficient in reaching the optimum as it moved irregularly in the solution space. SSA displayed a low exploration capacity due to its excessive dependence on leader individuals, while EO reached the optimum solution more slowly due to its structure based on balance principles. The EO algorithm showed a moderate performance but had some difficulties in reaching the global optimum. The MPA was relatively successful, especially in hardness optimization, but was not as effective as other powerful algorithms in terms of electrical conductivity. Although the MFO algorithm features a chaotic search mechanism, it explored the solution space insufficiently and showed a tendency to becoming stuck in local minima. Although MVO has the capacity to search for alternative solutions, it showed lower performance in terms of suitability for the problem space in this study.

As a result of the foregoing, the success level of these algorithms is directly related to the suitability of the mechanisms they use while searching through the problem space. In particular, the dynamic adaptation capabilities of FDA and AHA show that they provide more advantages when handling complex material optimization problems. In this study, cold deformation and aging processes were modeled in direct relation to the precipitation kinetics in the microstructure. The use of metaheuristic algorithms provided a significant advantage in terms of materials engineering by enabling the optimization of these processes. This research proves that the most suitable material properties can be determined by modeling mechanical processes with metaheuristic optimization.

Another problem investigated under this study was the manner in which the hardness and electrical conductivity properties of the Cu-4Ti alloy changed over the course of undeformed and aged as well as deformed and aged processes. What was observed in the experiments is that the deformed samples could reach higher hardness values depending on the aging process, but there was a certain decrease in electrical conductivity. This change is directly related to precipitation kinetics, dislocation density, and microstructural transformations. The fact that hardness and conductivity values change depending on different parameter combinations in a wide experimental space complicates this optimization problem. At this point, metaheuristic algorithms play a critical role in determining the most adequate parameters by taking the minimum energy into account in this experimental space. Highly successful algorithms such as FDA and AHA have been able to optimize mechanical and electrical properties by creating the delicate balance between hardness and electrical conductivity. This optimization process reduces experimental costs and time loss while minimizing energy consumption and offering a sustainable production approach. Specifically, the wide search capabilities of metaheuristic algorithms are the sources of great advantages while seeking the optimum solution in these multivariable systems. This study shows that metaheuristic optimization methods offer an enormous potential when applied to the design of engineering materials with the most suitable mechanical and electrical properties.

## 4. Conclusions

The goal of this study was to systematically investigate the effects of cold deformation following solution treatment on the electrical conductivity and hardness of the precipitation-heat-treatable Cu-4Ti alloy under various conditions and for various aging duration times. The synergy between experimental analysis and metaheuristic modeling has provided an in-depth understanding of microstructural formation and the resulting mechanical and electrical properties.

The findings suggest that aging time and temperature have considerable influence on the behavior of the Cu-4Ti precipitates, and specifically on the alloy’s electrical conductivity and hardness. The microstructural evidence for the precipitates of Cu_4_Ti is precisely in the hardening, whereas the precipitate’s structure and content affect its electrical conductivity and electron scattering. The response in hardness is enhanced by prior cold deformation, thus increasing sites for precipitation. Conversely, high cold work reduces the ultimate electrical conductivity by leading to lattice strain, and consequently, electric flow is impaired.

Metaheuristic optimization was proved to predict and optimize aging conditions to provide good combinations of desirable properties. The modeling methodology in question successfully describes the intricacies of the interactions between parameters in processing, ensuring that the predictions of mechanical and electrical properties are accurate. The methodology is based on solid foundations which enable engineering alloys to be designed and optimized specifically for advanced engineering applications.

Polynomial regression analysis and integration with metaheuristic algorithms demonstrated high accuracy in the optimization of aging parameters and greatly enhanced the reliability and predictability of the properties of the Cu-4Ti alloy. This study not only explains the crucial influence of cold deformation and aging treatments on the microstructure evolution but also provides an efficient computational tool that may be implemented to solve larger alloy design problems. Future research may extend these computational models by adding other processing variables or by using advanced machine learning methodologies, thus expanding their application to other industries including aerospace, automotive, and electronics manufacturing.

Overall, this study makes strong contributions to the understanding of the microstructure–processing–property relationships in the Cu-4Ti alloys. Its findings not only provide an explicit methodology for optimizing the performance of materials, but also reveal the capabilities of computational intelligence which may be of use for the designing of alloys. The approach proposed under the study is universally applicable to any precipitate strengthening system of alloys, and as such, it offers an efficient and convenient methodology for designing future-generation, high-performance materials.

The main objective underpinning this study was to establish a practical and predictable relationship between process parameters, such as time and temperature, and the resulting material properties. Since physical modeling usually requires extensive experimental datasets, knowledge of phase transformation kinetics and detailed thermodynamic databases are considered important. Used at this stage, empirical models represent a more feasible and faster method. Furthermore, this approach also facilitates industry-oriented process optimization.

Future research needs to explore the use of other alloying components and their interactions with deformation and aging treatments to further optimize the mechanical and electrical properties of Cu-Ti alloys, which may potentially be supported by advanced computational models and artificial intelligence-based solutions. The tool of metaheuristic analysis can be utilized in the future to investigate the corrosion and wear resistance behavior of alloyed copper [75].

## Figures and Tables

**Table 1:** Chemical composition of the Cu-4Ti alloy (weight %).

Cu	Ti	Zn	P	Pb	Sn	Mn	Ni	Sb	Bi	As	Cd
95.83	3.95	0.13	0.065	0.003	0.009	0.030	0.01	0.001	0.001	0.001	0.001

**Table 2:** Heat treatment conditions applied to the investigated alloy.

Aging Temperature	Aging Time	Condition			
(T, °C)	(t, min)	Undeformed and Aged		Deformed and Aged	
		Electrical Conductivity	Hardness	Electrical Conductivity	Hardness
		(MS/m)	(HV5)	(MS/m)	(HV5)
450	1	3.1	137	3.4	221
	10	5.3	189	-	-
	15	5.8	202.3	5.1	292
	30	6.6	223	6.8	297
	60	7	229	7.6	296
	120	7.5	236	8.1	298
	420	8.4	248	9.2	283
500	1	3.1	147	3.6	225
	10	6	215	-	-
	15	6.4	219	5.3	300
	30	6.8	226	7	287
	60	7.2	236	8.9	279
	120	7.7	244	9.1	263
	420	8.9	183	9.5	240
550	1	3	132	4.4	230
	10	6.3	228	-	-
	15	6.5	228	6.5	273
	30	6.6	235	6.9	245
	60	7	218	7.7	218
	120	7.7	189	9.4	160
	420	8.9	178	11	157
600	1	4	135	3.7	256
	10	6.2	277	-	-
	15	6.3	259	7.4	251
	30	6.8	187	8	167
	60	7.5	177	8.3	162
	120	7.4	178	9.3	158
	420	7.0	170	8.5	152

**Table 3:** Results of the hardness (HV) and electrical conductivity [MS/m] tests after solutionizing (values averaged from 10 measurements).

Alloy	HV0.5	γ [MS/m]
CuTi4	125	2.80

**Table 4:** Hyperparameters of the metaheuristic algorithms used.

Algorithm	Key Hyperparameters	Typical Values/Range
Artificial Hummingbird Algorithm (AHA)	Migration coefficient	2n, n: population size
	Guided foraging probability	0.5
	Territorial foraging factor (b)	*N*(0,1)
	Guided factor (a)	*N*(0,1)
Ant Lion Optimizer (ALO)	Convergence factor (α)	1 to 2 (reduces over iterations)
	Elite selection probability	0.5
Atom Search Optimization (ASO)	Depth weight (α)	50
	Multiplier weight (β)	0.2
Equilibrium Optimizer (EO)	Exploration controlling (a_1_)	2
	Exploitation controlling (a_2_)	1
	Generation probability (GP)	0.5
	Randomization parameter (r)	Uniformly distributed in [0, 1]
Flow Direction Algorithm (FDA)	Flow velocity	0.1–2.0
	Number of neighbor (β)	1
Moth Flame Optimization (MFO)	Flame update mechanism	Logarithmic
	Spiral constant	1
	Convergence constant	[−1, −2]
Marine Predator Algorithm (MPA)	FAD effect	0.6
	Constant value (P)	0.8
	Random walk model	Lévy flight/Brownian motion
Multi-Verse Optimizer (MVO)	Wormhole existence probability (WEP)	0.2–1.0
	Traveling distance rate (TDR)	Decrease from 0.6 to 0
Particle Swarm Optimization (PSO)	Inertia weight	0.2–0.9
	Cognitive coefficient	2
	Social coefficient	2
Salp Swarm Algorithm (SSA)	Coefficient c_1_	Decrease from 0.2 to 0
	c_2_ and c_3_	Uniformly distributed in [0, 1]

**Table 5:** The optimum temperature and time parameters derived using metaheuristic algorithms to obtain the desired electrical conductivity and hardness values.

**Data Code**	**Desired Outputs**		**AHA**		**ALO**		**ASO**		**EO**		**FDA**	
	**Electrical Conductivity (EC)**	**Hardness (H)**	**Opt. Aging Temp.**	**Opt. Aging Time**	**Opt. Aging Temp**	**Opt. Aging Time**	**Opt. Aging Temp.**	**Opt. Aging Time**	**Opt. Aging Temp.**	**Opt. Aging Time**	**Opt. Aging Temp.**	**Opt. Aging Time**
	**MS/m**	**HV5**	**T °C**	**t min**	**T °C**	**t min**	**T °C**	**t min**	**T °C**	**t min**	**T °C**	**t min**
D1	4	150	569.9	1.9	450.0	2.6	552.3	1.9	569.9	1.9	569.9	1.9
D2	8.9	183	452.2	7.6	450.0	7.9	497.7	5.1	450.0	7.9	450.0	7.9
D3	5	190	575.6	5.1	493.2	6.2	516.4	5.7	493.5	6.1	496.7	6.1
D4	6.6	223	479.7	16.6	450.0	49.2	476.8	17.7	479.6	16.6	479.7	16.6
D5	7	230	486.6	23.4	486.6	40.0	488.5	26.0	486.6	23.4	486.6	23.4
D6	6.6	235	517.0	15.3	517.0	39.0	510.8	16.4	517.0	15.3	517.0	15.3
D7	6.3	260	599.1	14.0	599.9	13.9	580.1	17.3	600.0	13.8	600.0	13.8
D8	6.2	277	478.1	120.0	478.1	120.0	481.5	120.0	478.1	120.0	478.1	120.0
D9	8.3	162	600.0	31.1	600.0	31.1	586.7	39.5	600.0	31.1	600.0	31.1
D10	7.5	220	576.8	23.1	536.7	49.7	533.3	59.9	533.2	60.9	533.2	60.8
D11	7	245	531.1	35.0	537.1	31.3	529.6	36.1	531.2	35.0	531.1	35.0
D12	4	256	462.2	7.5	462.9	7.5	482.4	7.3	462.0	7.5	462.0	7.5
D13	9	263	502.3	62.8	502.2	60.1	501.8	59.2	502.3	62.8	502.3	62.8
D14	8.9	279	486.3	63.2	485.6	61.9	483.0	57.1	486.2	62.9	486.3	63.2
D15	5.4	290	450.0	19.9	450.0	19.9	453.8	20.1	450.0	19.9	450.0	19.9
**Data Code**	**Desired Outputs**		**MFO**		**MPA**		**MVO**		**PSO**		**SSA**	
	**Electrical Conductivity (EC)**	**Hardness (H)**	**Opt. Aging Temp.**	**Opt. Aging Time**	**Opt. Aging Temp.**	**Opt. Aging Time**	**Opt. Aging Temp.**	**Opt. Aging Time**	**Opt. Aging Temp.**	**Opt. Aging Time**	**Opt. Aging Temp.**	**Opt. Aging Time**
	**MS/m**	**HV5**	**T °C**	**t min**	**T °C**	**t min**	**T °C**	**t min**	**T °C**	**t min**	**T °C**	**t min**
D1	4	150	569.9	1.9	569.9	1.9	568.9	1.9	569.9	1.9	557.4	1.9
D2	8.9	183	450.0	7.9	450.0	7.9	450.5	7.8	579.4	7.9	450.0	7.8
D3	5	190	512.9	5.8	535.4	5.6	571.2	6.0	493.4	6.2	509.1	5.8
D4	6.6	223	475.6	18.2	479.7	16.6	450.0	49.2	479.7	16.6	450.0	49.2
D5	7	230	489.6	28.3	486.6	23.4	492.4	30.9	486.6	23.4	496.7	37.4
D6	6.6	235	515.9	16.0	517.0	15.3	517.0	15.3	517.0	15.3	517.0	15.3
D7	6.3	260	598.9	14.1	600.0	13.8	598.9	14.0	599.2	14.0	599.9	13.9
D8	6.2	277	478.1	120.0	478.1	120.0	600.0	20.2	600.0	20.2	600.0	20.2
D9	8.3	162	600.0	31.1	600.0	31.1	600.0	31.1	575.5	51.6	600.0	31.1
D10	7.5	220	533.2	60.5	583.5	20.5	537.5	48.4	533.0	61.8	533.1	61.0
D11	7	245	534.8	32.6	531.1	35.0	526.9	38.3	530.9	35.1	530.9	35.1
D12	4	256	454.1	7.2	462.0	7.5	462.7	7.5	518.2	6.3	465.2	7.5
D13	9	263	502.3	62.4	502.3	62.8	503.5	56.7	502.2	62.1	502.6	61.9
D14	8.9	279	485.7	62.1	486.3	63.2	484.7	60.3	485.1	61.0	485.4	61.4
D15	5.4	290	450.0	19.9	450.0	19.9	450.0	19.9	461.2	21.0	450.0	19.9

## Data Availability

The original contributions presented in this study are included in the article. Further inquiries can be directed to the corresponding author.

## References

[1] Huang L., Peng L., Mi X., Zhao G., Huang G., Xie H., Zhang W. (2022). Effect of Cold Working on the Properties and Microstructure of Cu-3.5 wt% Ti Alloy. Materials.

[2] Dölling J., Kuglstatter M., Prahl U., Höppel H.W., Ortner P., Ott B., Kracun S.F., Fehlbier M., Zilly A. (2024). Analyzing the Precipitation Effects in Low-Alloyed Copper Alloys Containing Hafnium and Chromium. Metals.

[3] Mofrad H.E., Raygan S., Forghani B.A., Hanaei K., Ahadi F. (2012). Effect of cold-working and aging processes on the microstructure, mechanical properties and electrical conductivity of Cu–13.5%Mn–4%Ni–1.2%Ti alloy. Mater. Des..

[4] Hang F., Gao T., Gao J., Qin L., Meng X., Zhang M., Ling H., Zhong J., Zhang L. (2024). Breaking hardness and electrical conductivity trade-off in Cu-Ti alloys through machine learning and Pareto front. Mater. Res. Lett..

[5] Nagarjuna S., Balasubramanian K., Sarma D.S. (1999). Effect of prior cold work on mechanical properties, electrical conductivity and microstructure of aged Cu-Ti alloys. J. Mater. Sci..

[6] Valiev R., Langdon T. (2006). Principles of equal-channel angular pressing as a processing tool for grain refinement. Prog. Mater. Sci..

[7] Yang H.B., Chai Y.F., Jiang B., He C., Yang Q.S., Yaun M. (2022). Enhanced mechanical properties of Mg-3Al-1Zn alloy sheets through slope extrysion. Int. J. Miner. Metall. Mater..

[8] Tsuji N., Saito Y., Lee S., Minamino Y. (2003). ARB (Accumulative Roll-bonding) and other new techniques to produce bulk ultrafine grained materials. Adv. Eng. Mater..

[9] Zhilyaev A., Langdon T. (2008). Using high-pressure torsion for metal processing: Fundamentals and applications. Prog. Mater. Sci..

[10] Wang P.F., Liang M., Xu X.Y., Feng J.Q., Li C.S., Zhang P.X., Li J.S. (2021). Effect of groove rolling on the microstructure and properties of Cu-Nb microcomposite wires. Int. J. Miner. Metall. Mater..

[11] Li R., Xiao Z., Li Z., Meng X., Wang X. (2023). Work Hardening Behavior and Microstructure Evolution of a Cu-Ti-Cr-Mg Alloy during Room Temperature and Cryogenic Rolling. Materials.

[12] Rahman M.S., Joy R.A. (2024). Analysis of Microstructure, Recrystallization, and Hardness of Copper Metal Based on Cold Working and Annealing Processes. Int. J. Mater. Sci. Eng..

[13] Jo M., Choi E.A., Ahn J.H., Son Y.G., Kim K., Lee J., Semboshi S., Han S.Z. (2019). Effect of prior cold working before aging on the precipitation behavior in a Cu-3.5 wt% Ti alloy. J. Korean Inst. Met. Mater..

[14] Xin G., Zhou M., Jing K., Hu H., Li Z., Zhang Y., Bai Q., Tian C., Tian B., Li X. (2024). Heat treatment effects on microstructure and properties of Cu–Ti–Fe alloys. Mater. Sci. Eng. A.

[15] Nagarjuna S., Balasubramanian K., Sarma D.S. (1995). Effects of Cold Work on Precipitation Hardening of Cu-4.5 mass%Ti Alloy. Materials Transactions. Mater. Trans. JIM.

[16] Markandeya R., Nagarjuna S., Sarma D.S. (2005). Effect of prior cold work on age hardening of Cu–4Ti–1Cr alloy. Mater. Sci. Eng. A.

[17] Wang X., Xiao Z., Zhou T., Jiang X. (2024). Deformation mechanism and properties evolution of a copper alloy with ultra-high strength after cold drawing and subsequent aging treatment. Mater. Sci. Eng. A.

[18] Tu Y., Liu X., Wang W., Zhang W., Feng Q. (2022). Deformation-aging behavior and property evolution of Cu–Ti alloys prepared by accumulative roll bonding-deformation diffusion process. Mater. Sci. Eng. A.

[19] Semboshi S., Amano S., Fu J., Iwase A., Takasugi T. (2017). Kinetics and Equilibrium of Age-Induced Precipitation in Cu-4 At. Pct Ti Binary Alloy. Met. Mater. Trans. A.

[20] Li C., Wang X., Li B., Shi J., Liu Y., Xiao P. (2020). Effect of cold rolling and aging treatment on the microstructure and properties of Cu–3Ti–2Mg alloy. J. Alloys Compd..

[21] Eze A.A., Jamiru T., Sadiku E.R., Mondiu Ọ.D., Kupolati W.K., Ibrahim I.D., Obadele B.A., Olubambi P.A., Diouf S. (2018). Effect of titanium addition on the microstructure, electrical conductivity and mechanical properties of copper by using SPS for the preparation of Cu-Ti alloys. J. Alloys Compd..

[22] Yang K., Guo M., Wang H., Mo Y., Wang M., Liu F., Wang Y., Liang D., Lou H. (2025). Synergistical Effects of Mg Microalloying, Deformation, and Aging on the Strength and Electrical Conductivity of Cu-3.3 wt%Ti Alloy. Adv. Eng. Mater..

[23] Zhao Q., Yang H., Liu J., Zhou H., Wang H., Yang W. (2021). Machine learning-assisted discovery of strong and conductive Cu alloys: Data mining from discarded experiments and physical features. Mater. Des..

[24] Guo X., Li L., Liu G., Kang H., Chen Z., Guo E., Jie J., Wang T. (2024). Microstructure and properties of Cu-3Ti-0.3Cr-0.15Mg alloy designed via machine learning. Mater. Sci. Eng. A.

[25] Li L., Jie J., Guo X., Liu G., Kang H., Chen Z., Guo E., Wang T. (2025). Accelerated Composition-Process-Properties Design of Precipitation-Strengthened Copper Alloys Using Machine Learning Based on Bayesian Optimization. Mater. Res. Lett..

[26] Fotopoulos V., O’Hern C.S., Shattuck M.D., Shluger A.L. (2024). Modeling the Effects of Varying the Ti Concentration on the Mechanical Properties of Cu–Ti Alloys. ACS Omega..

[27] Babaheydari R.M., Mirabootalebi S.O., Akbari G.H., Salehifar H. (2023). Prediction of Hardness of Copper-based Nanocomposites Fabricated by Ball-milling using Artificial Neural Network. Int. J. Eng..

[28] Huang Z., Shi R., Xiao X., Fu H., Chen Q., Xie J. (2021). Mechanism investigation on high-performance Cu-Cr-Ti alloy via integrated computational materials engineering. Mater. Today Commun..

[29] Zhao W., Wang L., Mirjalili S. (2022). Artificial hummingbird algorithm: A new bio-inspired optimizer with its engineering applications. Comput. Methods Appl. Mech. Eng..

[30] Yang P., Li C., Qiu Y., Huang S., Zhou J. (2023). Metaheuristic Optimization of Random Forest for Predicting Punch Shear Strength of FRP-Reinforced Concrete Beams. Materials.

[31] Mehmood K., Chaudhary N.I., Khan Z.A., Cheema K.M., Raja M.A.Z. (2024). Atomic physics-inspired atom search optimization heuristics integrated with chaotic maps for identification of electro-hydraulic actuator systems. Mod. Phys. Lett. B.

[32] Kamalakkannan P., Kumar B.V., Kalamani M. (2024). Optimal nonlinear Fractional-Order Proportional-Integral-Derivative controller design using a novel hybrid atom search optimization for nonlinear Continuously stirred Tank reactor. Therm. Sci. Eng. Prog..

[33] Fan Y., Zhang S., Wang Y., Xu D., Zhang Q. (2023). An Improved Flow Direction Algorithm for Engineering Optimization Problems. Mathematics.

[34] Yiğit H., Ürgün S., Mirjalili S. (2022). Comparison of recent metaheuristic optimization algorithms to solve the SHE optimization problem in MLI. Neural Comput. Applic..

[35] Yu H., Quan J., Han Y., Heidari A.A., Chen H. (2024). An enhanced Moth-Flame optimizer with quality enhancement and directional crossover: Optimizing classic engineering problems. Artif. Intell..

[36] Ürgün S., Yiğit H., Mirjalili S. (2023). Investigation of Recent Metaheuristics Based Selective Harmonic Elimination Problem for Different Levels of Multilevel Inverters. Electronics.

[37] Aydemir S.B. (2023). Enhanced marine predator algorithm for global optimization and engineering design problems. Adv. Eng. Softw..

[38] Liu S., Qin H., Liu G., Qu Y., Tang Y., Jiang Z. (2024). A comprehensive opposition Multi-Verse Optimizer ensemble coordination constraint handling technique for hybrid hydro-thermal-wind problem. Expert Syst. Appl..

[39] Ürgün S., Yiğit H., Fidan S., Sınmazçelik T. (2024). Optimization of Laser Cutting Parameters for PMMA Using Metaheuristic Algorithms. Arab. J. Sci. Eng..

[40] Abualigah L., Shehab M., Alshinwan M., Alabool H. (2019). Salp swarm algorithm: A comprehensive survey. Neural Comput. Appl..

[41] Konieczny J., Labisz K., Ürgün S., Yiğit H., Fidan S., Bora M.Ö., Atapek Ş.H. (2025). Metaheuristics Algorithm-Based Optimization for High Conductivity and Hardness CuNi2Si1 Alloy. Materials.

[42] Poonia A., Kishor M., Ayyagari K.P.R. (2024). Designing of high entropy alloys with high hardness: A metaheuristic approach. Sci. Rep..

[43] Kolev M. (2024). Predictive Analysis of Mechanical Properties in Cu-Ti Alloys: A Comprehensive Machine Learning Approach. Modelling.

[44] Sikdar S., Mukherjee I. (2011). A Holistic Framework for Multiple Response Optimization of Hot Strip Rolling Process. Mater. Manuf. Process..

[45] (1925). International Standard of Resistance for Copper.

[46] Lomakin I., Castillo-Rodríguez M., Sauvage X. (2019). Microstructure, mechanical properties and aging behaviour of nanocrystalline copper–beryllium alloy. Mater. Sci. Eng. A.

[47] Gorsse S., Gouné M., Lin W.-C., Girard L. (2023). Dataset of mechanical properties and electrical conductivity of copper-based alloys. Sci. Data.

[48] Rdzawski Z. (2009). Miedź Stopowa, Wydawnictwo Politechniki Śląskiej.

[49] Moyson S., Vissenberg K., Fransen E., Blust R., Husson S.J. (2018). Mixture Effects of Copper, Cadmium, and Zinc on Mortality and Behavior of Caenorhabditis elegans. Environ. Toxicol. Chem..

[50] Rouxel B., Mischler S., Log’e R., Igual Munoz A. (2023). Wear behaviour of novel copper alloy as an alternative to copper-beryllium. Wear.

[51] Jakubowski M., Pałczyński C. (2015). Beryllium. Handbook on the Toxicology of Metals 4E.

[52] (2008). Regulation of the European Parliament and of the Council of 16 December 2008 on classification, labelling and packaging of substances and mixtures, amending and repealing Directives 67/548/EEC and 1999/45/EC, and amending Regulation (EC) No 1907/2006.

[53] Rdzawski Z., Stobrawa J. (1993). Thermomechanical processing of Cu–Ni–Si–Cr–Mg alloy Materials. Sci. Technol..

[54] Li Z., Li S., Xiao Z., Lei Q., Xing Y. (2015). CuTi-Series Elastic Copper Alloy and Preparation Method Thereof. Patent.

[55] Nagarjuna S., Srinivas M. (2002). High temperature tensile behaviour of a Cu-1.5 wt.% Ti alloy. Mater. Sci. Eng..

[56] Nagarjuna S., Sarma D.S. (1999). On the variation of lattice parameter of Cu solid solution with solute content in Cu-Ti alloys. Scr. Mater..

[57] Nagarjuna S., Srinivas M., Balasubramanian K., Sarma D.S. (1995). On the deformation characteristic of solution treated Cu-Ti alloys. Scr. Metall. Mater..

[58] Nagarjuna S., Srinivas M., Balasubramanian K., Sarma D.S. (1998). Effect of modulations on yield stress and strain. Scr. Mater..

[59] Semboshi S., Nishida T., Numakura H. (2011). Aging of Cu-3 at% Ti Alloys in Hydrogen Atmosphere: Influence of Hydrogen Pressure on Strength and Electrical Conductivity. Mater. Trans..

[60] Nagarjuna S., Srinivas M. (2008). Grain refinement during high temperature tensil testing of prior cold worked and peak aged Cu-Ti alloys: Evidence of superplasticity. Mater. Sci. Eng..

[61] Bzowski S., Gorczyca S. (1981). Phase transformation in Cu-Ti alloys. Metallurgy.

[62] Nagarjuna S., Chinta Babu U., Ghosal P. (2008). Effect of cryo-rolling on age hardening of Cu-1.5Ti alloy. Mater. Sci. Eng. A.

[63] Semboshi S., Al-Kassab T., Gemma R., Kirchheim R. (2009). Microstructural evolution of Cu-1 at% Ti alloy aged In a hydrogen atmosphere ant its relation with the electrical conductivity. Ultramicroscopy.

[64] Semboshi S., Nishida T., Numakura H. (2009). Microstructure and mechanical properties of Cu-3% at. Tialloy aged in a hydrogen atmosphere. Mater. Sci. Eng. A..

[65] Hameda A.A., Błaż L. (1998). Microstructure of hot-deformed Cu-3.45 wt.% Ti alloy. Mater. Sci. Eng. A..

[66] Hameda A.A., Błaż L. (1997). Flow softening during hot compression of Cu-3.45 wt.% Ti alloy. Scr. Mater..

[67] Cazac A.-M., Chelariu R.G., Cimpoesu R., Bernevig M.A., Benchea M., Jurca A.M., Radu A.M., Vasilescu G.D., Garaliu-Busoi B., Lupu F.C. (2024). Investigation of CuTi Alloy for Applications as Non-Sparking Material. Appl. Sci..

[68] Liu H., Zhang X., Yuan Z., Zhang X., Wang G., Qi F., Li C., Ding H., Liu Q. (2025). The influence of TiC on microstructure and properties of Cu–Ti alloy. Int. J. Refract. Met. Hard Mater..

[69] Hong N., Liao Y., Chen H., Zhou C., Xie W., Wang H., Yang B. (2024). Precipitation behavior and strengthening mechanism in a Cu-3.5Ti-0.1 Tm alloy. Mater. Sci. Eng. A.

[70] Wang X., Chen C., Guo T., Zou J., Yang X. (2015). Microstructure and Properties of Ternary Cu-Ti-Sn Alloy. J. Mater. Eng. Perform..

[71] Božić D., Dimčić O., Dimčić B., Cvijović I., Rajković V. (2008). The cmobination of precipitation and dispersion hardening In powder metallurgy produced Cu-Ti-Si alloy. Mater. Charact..

[72] Rdzawski Z., Stobrawa J., Głuchowski W., Konieczny J. (2010). Thermomechanical processing of CuTi4 alloy. J. Achiev. Mater. Manuf. Eng..

[73] Konieczny J. (2013). Forming of the structure and application properties of precipitation reinforced titanium copper. Open Access Libr. Sci. Int. J. Word Acad. Mater. Manuf. Eng. Int. OCSCO Word Press.

[74] Liao Y., Guo C., Zhou C., Xie W., Yang B., Wang H. (2023). Stability of the metastable β′-Cu4Ti phase in Cu–Ti alloys: Role of the Ti content. Mater. Charact..

[75] Konieczny J., Labisz K. (2021). Thermal Analysis and Selected Properties of CuNi2Si Alloy Used for Railway Traction. Materials.

